# The Behavioral and Neural Mechanisms Underlying the Tracking of Expertise

**DOI:** 10.1016/j.neuron.2013.10.024

**Published:** 2013-12-18

**Authors:** Erie D. Boorman, John P. O’Doherty, Ralph Adolphs, Antonio Rangel

**Affiliations:** 1 Department of Humanities and Social Sciences, California Institute of Technology, Pasadena, CA 91125, USA; 2 Computation and Neural Systems, California Institute of Technology, Pasadena, CA 91125, USA; 3 Centre for Functional Magnetic Resonance Imaging of the Brain, University of Oxford, John Radcliffe Hospital, Oxford OX3 9DU, UK

## Abstract

Evaluating the abilities of others is fundamental for successful economic and social behavior. We investigated the computational and neurobiological basis of ability tracking by designing an fMRI task that required participants to use and update estimates of both people and algorithms’ expertise through observation of their predictions. Behaviorally, we find a model-based algorithm characterized subject predictions better than several alternative models. Notably, when the agent’s prediction was concordant rather than discordant with the subject’s own likely prediction, participants credited people more than algorithms for correct predictions and penalized them less for incorrect predictions. Neurally, many components of the mentalizing network—medial prefrontal cortex, anterior cingulate gyrus, temporoparietal junction, and precuneus—represented or updated expertise beliefs about both people and algorithms. Moreover, activity in lateral orbitofrontal and medial prefrontal cortex reflected behavioral differences in learning about people and algorithms. These findings provide basic insights into the neural basis of social learning.

## Highlights

• We use our own likely actions and others’ explicit accuracy to track their expertise
• We do this differently when observing humans compared to algorithms
• Expertise beliefs and updates are tracked by brain regions linked to mentalizing
• lOFC and mPFC mediate differences in expertise learning for humans and algorithms

## Teaser

People learn about others’ expertise by considering their own likely actions and others’ accuracy but do this differently for humans and nonhuman agents. Boorman et al. show that key expertise learning signals are encoded by brain regions linked to theory of mind and contingent learning.

## Introduction

Is President Obama an expert? How about the colleagues down the hall? Whether assessing politicians or colleagues, we continually form and update impressions of others’ abilities. This skill carries considerable advantages because identifying the expertise of group members dramatically facilitates group performance in a range of contexts and is thought to enhance the survival fitness of social groups (Einhorn et al., 1977; Libby et al., 1987; Littlepage et al., 1997; Yetton and Bottger, 1982). Perceptions of expertise emerge by age eight (Henrich and Broesch, 2011) and appear to be key in guiding whom people select as political leaders, role models, professional advisors, employees, students, and colleagues (Aronson, 2003; Frith and Frith, 2012). Taken together, this suggests that tracking the ability or expertise of others is critical for effectively navigating our complex social world. Despite this, the computational and neurobiological basis of tracking others’ abilities is presently unknown.

Pioneering neuroscience studies on social learning have begun to reveal the neural mechanisms responsible for vicarious learning about the world (Burke et al., 2010; Cooper et al., 2012; Olsson and Phelps, 2007), as well as for learning about other agents’ beliefs, intentions, and expected future behavior (Behrens et al., 2008; Cooper et al., 2010; Hampton et al., 2008; Suzuki et al., 2012; Tomlin et al., 2006; Yoshida et al., 2010). However, the computational and neural underpinnings of learning about other agents’ attributes, such as their expertise, have received much less attention.

To effectively learn a person’s expertise in an uncertain world, our brains must assign causal responsibility for good or bad performance to their abilities, rather than to chance. Recent findings across species in the field of reinforcement learning have implicated lateral orbitofrontal cortex (lOFC), medial frontal and prefrontal cortex (MFC and mPFC, respectively), and dorsomedial striatum in aspects of contingent learning or credit assignment—the processes by which causal responsibility for a particular reward is attributed to a particular choice (Balleine et al., 2008; Noonan et al., 2011; Takahashi et al., 2011; Tanaka et al., 2008; Walton et al., 2010). It remains an open question whether similar or distinct neural systems underlie social contingent learning.

Another open question about expertise tracking concerns the nature of the learning mechanism. Because little is known about this, the set of potential learning mechanisms to be considered range from relatively simple algorithms, to relatively sophisticated ones based on optimal observer models. Recent findings have highlighted the prominence of simulation during executed and observed choice (Nicolle et al., 2012; Patel et al., 2012), as well as emulation learning (Suzuki et al., 2012). These studies suggest that subjects’ assessments of others’ expertise might depend upon their own simulated beliefs about the world.

Another critical open question in social learning concerns whether forming and updating beliefs about human and nonhuman agents involve distinct processes. To date, most computational accounts of social learning have lacked matched human and nonhuman comparisons (Behrens et al., 2008; Cooper et al., 2010; Hampton et al., 2008; Suzuki et al., 2012; Yoshida et al., 2010). Therefore, it is possible that some of the computations that have been attributed to learning specifically about other people are in fact also engaged when learning about nonhuman agents.

**Figure 1:**
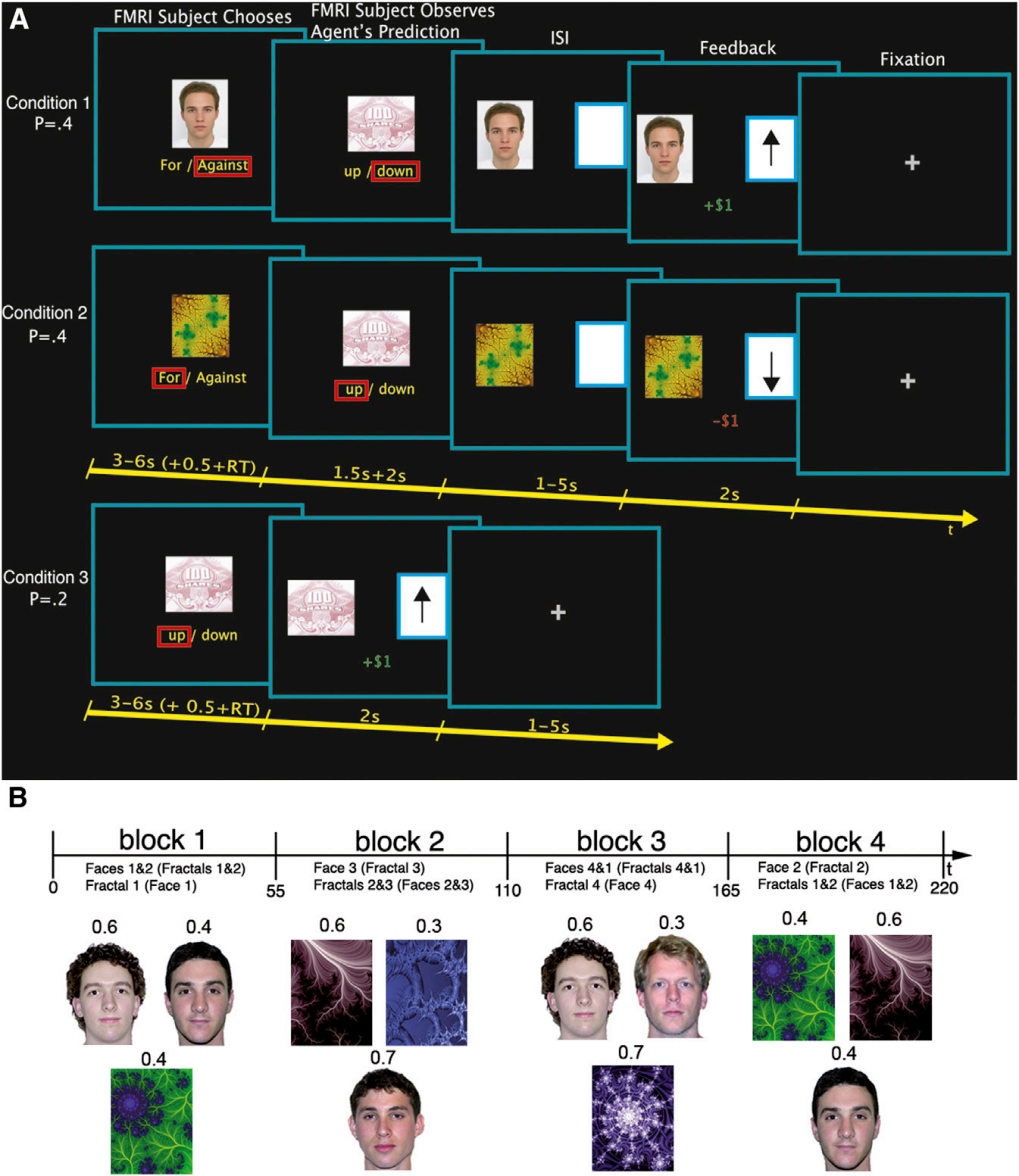
Experimental Task (A) Experimental task and timeline are shown. Participants were presented with either a picture of a human face (condition 1), a 2D fractal image symbolizing an algorithm (condition 2), or a hypothetical asset (condition 3). In conditions 1 and 2, subjects had to either bet for or against the agent. After a brief delay, they observed the agent’s choice: a prediction about whether the hypothetical asset would increase or decrease in value. Following a jittered fixation period, feedback was presented indicating whether the asset went up or down and whether the subject made or lost $1 for correct or incorrect predictions, respectively. In condition 3, the subject had to predict whether the asset would go up or down, and then received immediate feedback. ISI, interstimulus interval; RT, reaction time. (B) The task was divided into four blocks of 55 trials each. In each block, the subject observed the predictions of three agents (either two people and one algorithm, or the reverse). The true performance level of an agent is shown above each stimulus. Assignment of specific faces and fractal images to the corresponding predictions was pseudorandomly generated and counterbalanced across subjects.

We addressed these questions by designing an fMRI task that required human participants to form and update beliefs about the expertise of both people and algorithms through observation of their predictions in a simulated stock market (Figure 1). Crucially, participants’ expected monetary reward and reward prediction errors (rPEs) were carefully decorrelated from expertise estimates and expertise-updating signals.

Behaviorally, we found that a model-based sequential learning algorithm described subject choices better than several alternative models. Furthermore, when subjects believed that agents made the better choice, they effectively credited people more than algorithms for correct predictions and penalized them less for incorrect predictions. Neurally, we found that many components of the mentalizing network tracked or updated beliefs about the expertise of both people and algorithms. Finally, lOFC and mPFC activity reflected behavioral differences in social learning.

## Results

**Figure 2:**
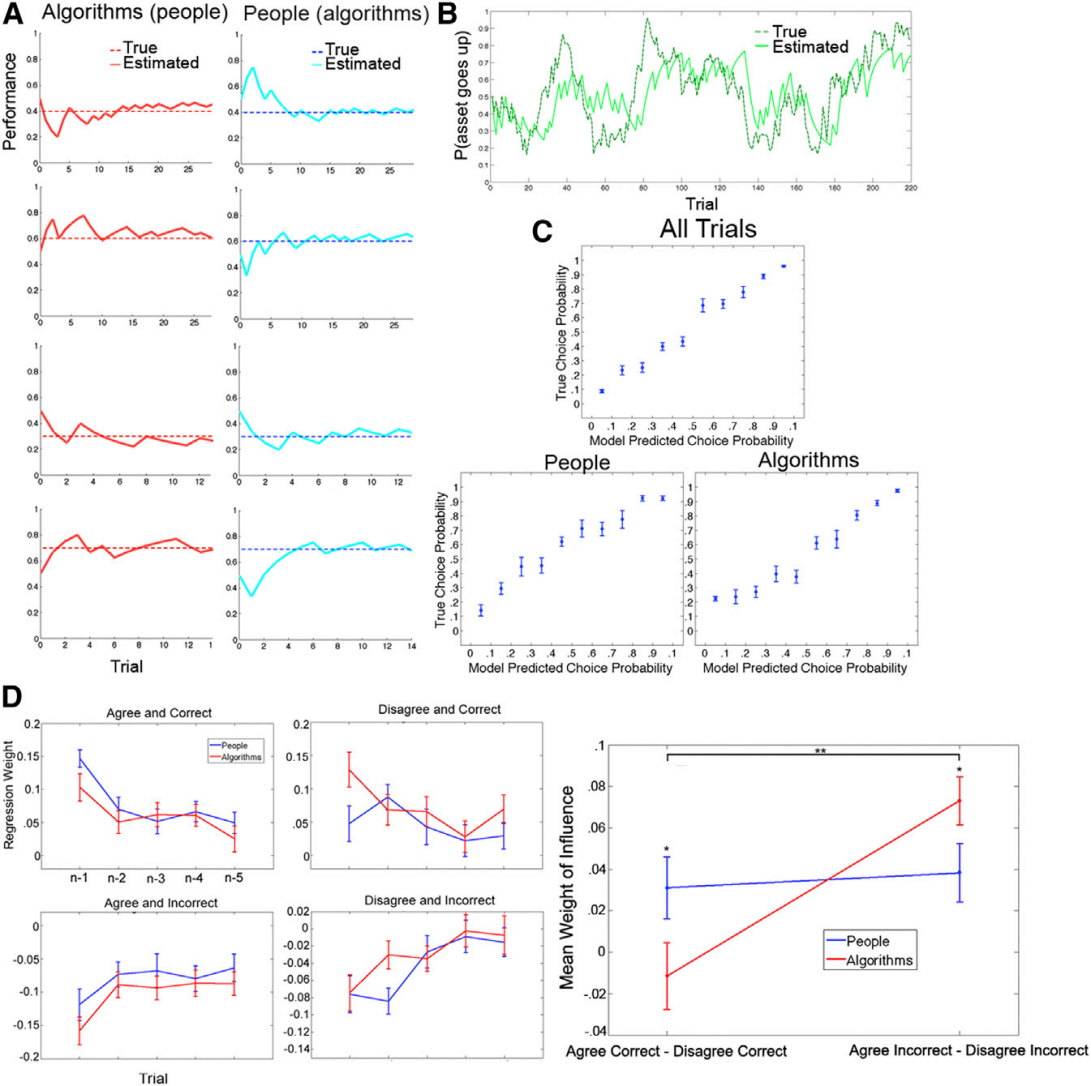
Task Parameters and Behavioral Analyses (A) True probabilities and model estimates of correct performance for the eight agents (four people and four algorithms) that subjects observed during the experiment are shown for one subject. For half of the subjects, blue represents people, and red represents algorithms; this was reversed for the other half, as indicated by parentheses. (B) Underlying probability that the asset’s value would increase and corresponding model estimates are plotted across trials. (C) Predictions of the best-fitting behavioral model are plotted against the true choice frequencies for all trials (top) and for predictions about people (bottom left) and algorithms (bottom right). Circles indicate means. Error bars represent ±SEM. (D) In the left panels, regression coefficients for correct and incorrect agent predictions of past trials are plotted but divided into correct trials with which subjects agree (Agree and Correct), disagree (Disagree and Correct), and incorrect trials with which subjects agree (Agree and Incorrect) and disagree (Disagree and Incorrect). In the rightmost panel, mean coefficients reflecting the overall influence of outcomes across trials n-1 to n-5 for correct trials with which subjects agree, compared to disagree (Agree Correct − Disagree Correct), and mean coefficients for incorrect trials with which subjects agree, compared to disagree (Agree Incorrect − Disagree Incorrect), are plotted separately for people (blue) and algorithms (red). Note that inverse coefficients are computed for incorrect trials such that the y axis indicates positive effects for correct trials and negative effects for incorrect trials. ^∗^p < 0.05; ^∗∗^p < 0.01. See also Figure S2.

To investigate how humans form and update impressions of other agents’ abilities, we scanned 25 participants while they made two different types of predictions: predictions about whether or not a specific agent (condition 1, person; condition 2, computer algorithm) would accurately predict increases or decreases in the value of a hypothetical financial asset (Figure 1A), and predictions about whether or not the financial asset would go up or down (condition 3; Figure 1A). Each agent had a fixed probability of predicting the asset’s movement accurately (Figure 2A), although this was not told to the subjects. As a result, the agents’ forecasting performance was independent of the asset’s performance. The asset increased or decreased in value on any particular trial with a drifting probability (Figure 2B). Subjects’ payoffs depended on the quality of their predictions, and not on the performance of the asset: every trial subjects won $1 for correct guesses and lost $1 for incorrect ones. See the Experimental Procedures for details.

### Behavior: Learning about the Asset

We assumed that subjects learned about the asset using a Bayesian model that allowed for estimates of the probability of price changes to evolve stochastically with changing degrees of volatility. This part of the model is based on previous related work on Bayesian learning about reward likelihood (Behrens et al., 2007, 2008; Boorman et al., 2011). The model described in the Supplemental Information (available online) learned to effectively track the performance of the asset, as shown in Figure 2B (Table S1). Furthermore, on average, it successfully predicted 80.0% (SE, 2.0%) of subjects’ asset predictions and dramatically outperformed a standard reinforcement-learning algorithm with a Rescorla Wagner update rule (Rescorla and Wagner, 1972) that allowed for subject-specific learning rates (see Table S1 and Supplemental Information for details).

### Learning about Ability: Model-Based Behavioral Analyses

We considered four natural classes of behavioral models according to which participants might form and update beliefs about the agents’ expertise (see Experimental Procedures and Supplemental Information for formal descriptions). All of the models assumed that subjects used information about agents’ performance to update beliefs about their ability using Bayesian updating. The models differed on the information that they used to carry out the updates, and on the timing of those updates within a trial. First, we considered a *full model* of the problem, given the information communicated to subjects, which uses Bayes rule to represent the joint probability distribution for the unknowns (i.e., the asset predictability and an agent’s ability), given past observations of asset outcomes and correct and incorrect guesses. This model predicts that subjects learn about the asset and agents together, on the basis of both past asset outcomes and the past performance of agents. This model would represent an optimal approach for a setting in which these two parameters fully governed agent performance. Second, we considered a *pure evidence model* in which subjects updated beliefs at the end of the trial based solely on the agent’s performance (i.e., whether the agent guessed the asset performance for the trial correctly). Importantly, this was done independently of whether or not the subject believed that the agent made the better choice, given the subject’s own beliefs about the asset. Third, we considered a *pure simulation model*, which does the converse. Here, the model predicts that the subject updates beliefs on the basis of whether or not the agent made the better choice according to the subject’s own beliefs about the asset and independently of the outcome at the end of the trial. In this case, the ability update takes place in the middle of the trial, when the agent’s choice is revealed. Finally, we considered a *sequential model* that effectively combines the updates of the evidence and simulation models sequentially. In this case, subjects update their ability estimates in the middle of the trial based on their belief about the quality of the agent’s choice and then update this new belief again at the end of the trial based on the performance of the agent’s prediction.

Out of all models tested, the Bayesian sequential model best matched subjects’ actual bets, as assessed by Bayesian information criterion (BIC; see Table 1), which penalizes additional free parameters. As described in the Supplemental Information, and reported in Table 1, we also tested several reinforcement-learning versions of these models, with different degrees of complexity. None of them performed as well as the Bayesian sequential model. Figure 2A depicts the predictions of the sequential model alongside the agent’s true probability of making correct predictions, which shows that the model was able to learn the agents’ expertise parameters quickly and accurately. Furthermore, comparison of actual choice frequencies with the predictions of the sequential model revealed a good fit both across all trials and when considering predictions about people and algorithms separately (Figure 2C). See Figure S1 for a comparison of model fit by subject.

### Learning about Ability: Regression-Based Behavioral Analyses

Interestingly, the optimal inference model in conditions 1 and 2 is the pure evidence one, where all updating takes place at the end of the trial based on the correctness of agents’ guesses. This is because agent expertise is given by a constant probability of guessing the direction of asset price change correctly, independent of actual asset performance. Because the sequential model provides a superior fit to subjects’ choices, this implies that subjects’ behavior is not fully optimal for the task.

In order to explore the source of this deviation from task optimality, we carried out the following regression analysis. We predicted current bets on the basis of previous correct and incorrect predictions from the past five trials with a particular agent. See the Supplemental Information for details. As expected, subjects were more likely to bet for both specific people and algorithms following previous correct predictions by that specific agent, with the size of the effect diminishing over time (Figure S2A). To quantify the influence of past prediction outcomes on current choices, we computed the mean of the regression coefficients for the past five trials observing an agent for people and algorithms separately. When considering all trial types together, there were no differences between people and algorithms (t(24) < 1; p > 0.1).

However, an interesting difference between learning about people and algorithms emerged when guesses were divided into those with which subjects likely agreed, compared to those with which they disagreed (i.e., when subjects believed agents chose the better option, as inferred from the asset-tracking model). Subjects were more likely to bet for human agents following correct predictions with which they agreed than following correct predictions with which they disagreed (t(24) = 2.1; p < 0.05; Figure 2D). This “agreement boost” for correct trials was not present for algorithms (t(24) < 1; p > 0.1) and was larger for people than algorithms (t(24) = 2.0; p < 0.05). In addition, there was an effect of agreement on betting for people following incorrect trials (t(24) = 2.75; p < 0.01; Figure 2D), and this effect was even larger for algorithms (t(24) = 6.29; p < 0.0001; difference between people and algorithms, t(24) = −2.06 and p < 0.05). This interaction can be illustrated by plotting the mean regression coefficients from trial n-1 to n-5 for trials on which subjects would likely agree (A) compared to disagree (D) on correct (C) trials and incorrect (I) trials separately (Figure 2D). Formal tests of this difference revealed a significant interaction between outcome type (AC−DC versus AI−DI) and agent type (people versus algorithms; F(24,1) = 7.65; p < 0.01). Similar results were obtained when we simply computed choice frequencies following AC, DC, AI, and DI outcome types on trial n-1 (Figure S3). Together, the data indicate that human, but not algorithm agents, receive a boost from making correct predictions that agree with subjects’ beliefs about the asset. They also show that algorithms are penalized more than human agents for making incorrect predictions that disagree with the subjects’ beliefs about the asset.

We carried two robustness tests of this result. First, we tested if this interaction effect depended on the amount of experience. To do this, we repeated the regression analyses separately in the first and the second half of trials and found a significant interaction effect in both halves of the experiment (first half, t(24) = 1.75 and p = 0.046; second half, t(24) = 2.58 and p = 0.008), and no significant difference between them (t(24) = 0.11; p = 0.46). Second, we tested if the effect differed across blocks. This revealed no evidence for an interaction between the effects of trial type (AC, DC, AI, and DI) and block (first or second; all t(24) < 1.0; p > 0.1), suggesting that combining data across blocks was not problematic.

The regression analyses we have reported are complementary to the model-based fitting approach. The sequential model predicts that participants update their beliefs partly on the basis of agreement between the subject and agent, and partly on the basis of the agent’s correctness, but it does not allow for an interaction between the two. In a post hoc effort to directly relate these two approaches, we constructed an additional reinforcement-learning algorithm that allows for differential updating on AC, DC, AI, and DI trial types for people and algorithms (see Supplemental Information for details). Due to the large number of parameters, this model was not identifiable in individual subjects but could be identified for the group using a fixed effects analysis. We computed maximum likelihood estimates (MLEs) on the eight relevant learning rates: people, γ^p^ on AC trials, η^p^ on DC trials, φ^p^ on AI trials, and λ^p^ on DI trials; algorithms, γ^a^ on AC trials, η^a^ on DC trials, φ^a^ on AI trials, and λ^a^ on DI trials. As shown in Figure S4A, this analysis revealed a greater MLE for γ^p^ than for γ^a^, the learning rate constants on AC trials, but a smaller MLE for φ^p^ than φ^a^, the learning rate constants on AI trials. The differences between MLEs on DC and DI trials were notably smaller. These results are consistent with the regression results, in that the group of subjects updated their ability estimates more for people than algorithms following correct predictions with which they agreed but less for people than algorithms following incorrect predictions with which they disagreed.

### Neural Representation of Expected Value and rPE

We began the analysis of the fMRI data by searching for expected value (EV) signals at choice, and rPE signals at feedback. On the basis of previous findings, we predicted to find EV signals in ventromedial prefrontal cortex (vmPFC) at the time subjects made decisions and rPEs in striatum at the time of outcome (Boorman et al., 2009; FitzGerald et al., 2009; Klein-Flügge et al., 2011; Li and Daw, 2011; Lim et al., 2011; O’Doherty et al., 2004; Tanaka et al., 2004). At the time of decision, EVs are high when subjects believe that the agent will bet correctly or incorrectly with high probability because they can forecast their behavior confidently and low when they believe that the agent’s ability is close to 0.5 because they cannot.

**Figure 3:**
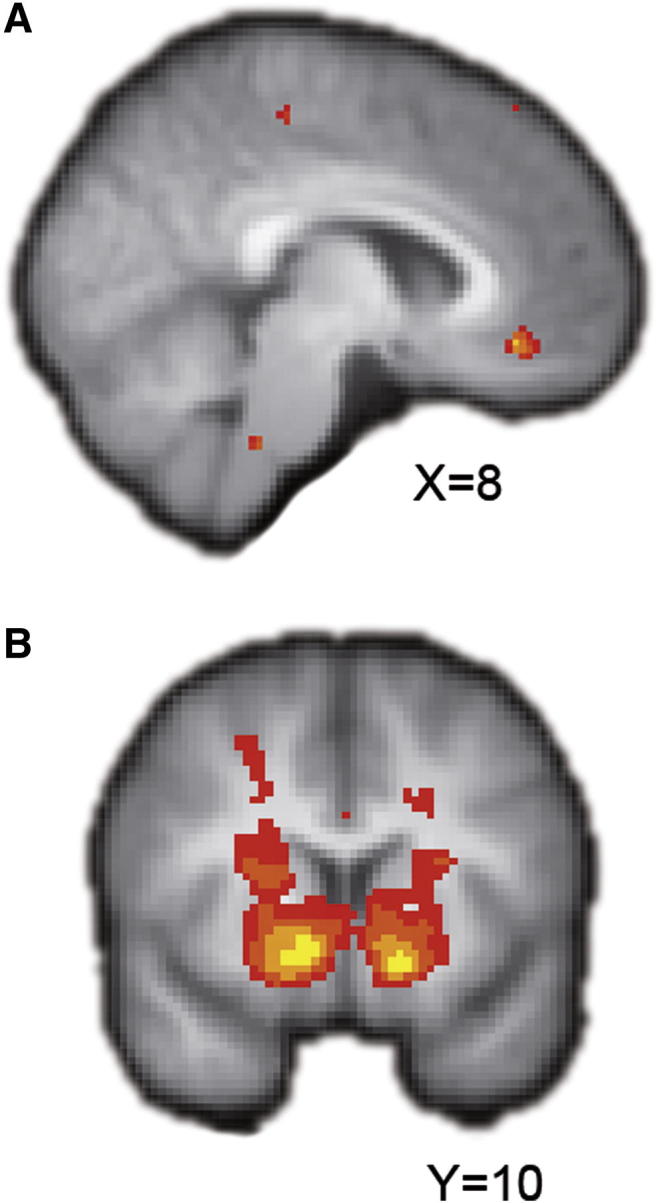
Expected Value and Reward Prediction Errors (A) Z-statistic map of the chosen option’s expected reward value at decision time is presented. (B) The same is shown for rPEs at feedback time. Maps are thresholded at Z > 3.1, p < 0.001, uncorrected for display purposes and are overlaid onto an average of subjects’ T1-weighted structural maps. Activations range from red (minimum) to yellow (maximum) Z-statistic values.

We estimated subjects’ trial-by-trial reward expectation and rPEs across all conditions using the sequential model and regressed these against the BOLD response across the whole brain. These contrasts revealed positive effects of the EV of the chosen option in vmPFC at choice and rPE at feedback in both ventral and dorsal striatum, among other regions (Figure 3; chosen value, Z > 3.1 and p < 0.001, voxel-wise thresholding; rPE, Z = 3.1 and p = 0.0l, corrected for multiple comparisons with cluster-based thresholding; Table S2). This provides evidence that activity in vmPFC and striatum reflects expected reward and rPEs in the context of our task and also provides further evidence for the descriptive validity of the sequential model.

### Neural Signatures of Ability and Ability Prediction Errors

The remaining analyses focus on identifying signals associated with computations that can support the learning and tracking of expertise. The logic of these tests is as follows. The sequential model makes three general predictions regarding the representation and updating of ability beliefs: (1) estimates of ability should be encoded at the time of decision making in order to guide subjects’ choices, (2) information related to simulation-based updates should be evident at the time the subject observes the agent’s prediction, and (3) information related to evidence-based updates should be evident at the time of feedback. To dissociate these signals from reward expectation and rPEs, we included expertise estimates (at decision), simulation-based expertise prediction errors (at the observed agent’s prediction), and evidence-based expertise prediction errors (at feedback) within the same general linear model (GLM) of the BOLD response as these reward terms. See the Experimental Procedures for details and Figure S5 for the correlation matrix between task variables. Importantly, we used unsigned prediction errors (i.e., the absolute value of prediction errors) as our marker of updating activity. The reason for this, which is explained in more detail in the Discussion, is that Bayesian updating is generally largest when outcomes deviate from expectations (i.e., when agents are surprised), and unsigned prediction errors provide a simple measure of such deviations.

### Expertise Estimates at Decision

**Figure 4:**
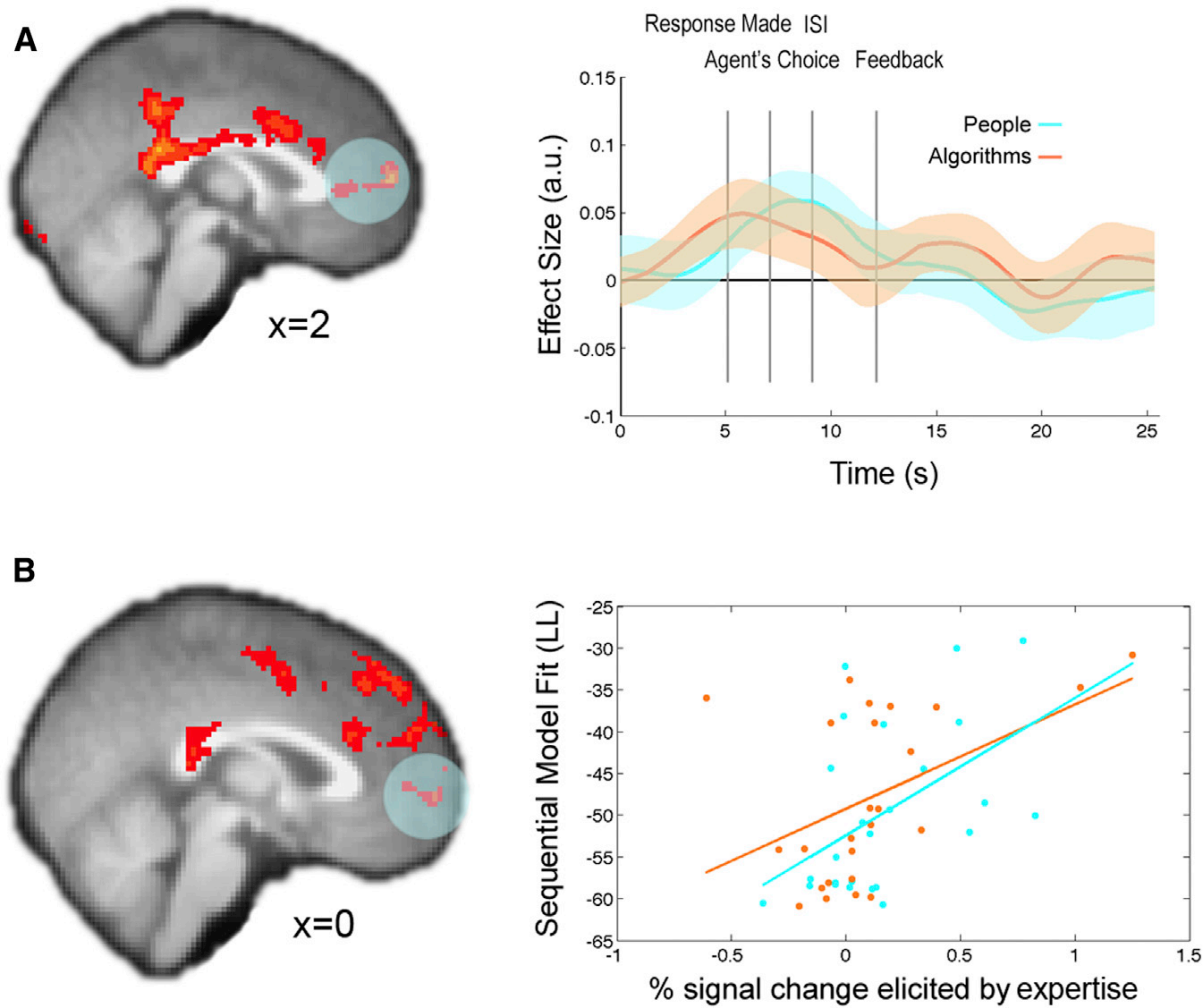
BOLD Effects of Ability Estimates (A) Left view shows a sagittal slice through the Z-statistic map (p = 0.05, cluster corrected across the whole brain) for ability belief, as predicted by the sequential model, independent of agent type (people or algorithms), at decision time. Right view shows the time course of the effect of expertise from independently identified rmPFC ROIs (circled), plotted separately for people (cyan) and algorithms (orange) across the entire trial. Dark lines indicate mean effects; shadows show ±SEM. (B) Left view is a sagittal slice through Z-statistic map (p = 0.05 whole-brain cluster corrected) relating to individual differences in the effect of expertise and the fit to behavior of the sequential model. In the right view, a scatterplot of the percent signal change elicited by expertise in independently identified rmPFC ROIs (circled) is plotted against the model fit (less negative numbers indicate better fit) for people (cyan) and algorithms (orange) separately. Activations range from red (minimum) to yellow (maximum) Z-statistic values.

We tested for correlates of subjects’ trial-by-trial ability estimates, independently of agent type (people or algorithms), using a whole-brain analysis. This analysis revealed a network of brain regions exhibiting positive effects of subjects' ability estimates, which included rostromedial prefrontal cortex (rmPFC), anterior cingulate gyrus (ACCg), and precuneus/posterior cingulate cortex (PCC) (Figure 4A; Z = 2.3, p = 0.05 whole-brain corrected; Table S2). Throughout the paper, we identify ROIs for further analysis in a way that avoids the potential for selection bias, by using the leave-one-out procedure described in the Supplemental Information. Inspecting the time course of the effects of ability for people and algorithms separately revealed similar response profiles that occurred specifically at decision time (Figure 4A). Notably, no regions showed significant differences in the neural response to expertise estimates for people and algorithms.

If our behavioral model accurately predicts subject choices, and our fMRI model identifies a neural representation of a crucial decision variable from the behavioral model, then one would expect a particularly strong neural effect of this variable in those subjects in whom the behavioral model provides a better description. Hence, we tested whether the fit of the sequential model to subject behavior was correlated with the BOLD response to ability in a between-subjects whole-brain analysis. This identified similar regions of rmPFC, ACCg, and PCC as the initial analysis and additionally a cluster in dorsomedial prefrontal cortex (dmPFC) (Figure 4B; Z = 2.3, p = 0.05 corrected; Table S3). Furthermore, analysis of independently identified ROIs in rmPFC demonstrated a significant correlation between the sequential model’s fit to a subject’s behavior and the neural effect of expertise for both people (r = 0.49; p = 0.01) and algorithms (r = 0.54; p < 0.01; Figure 4B).

### Simulation-Based Expertise Prediction Errors

**Figure 5:**
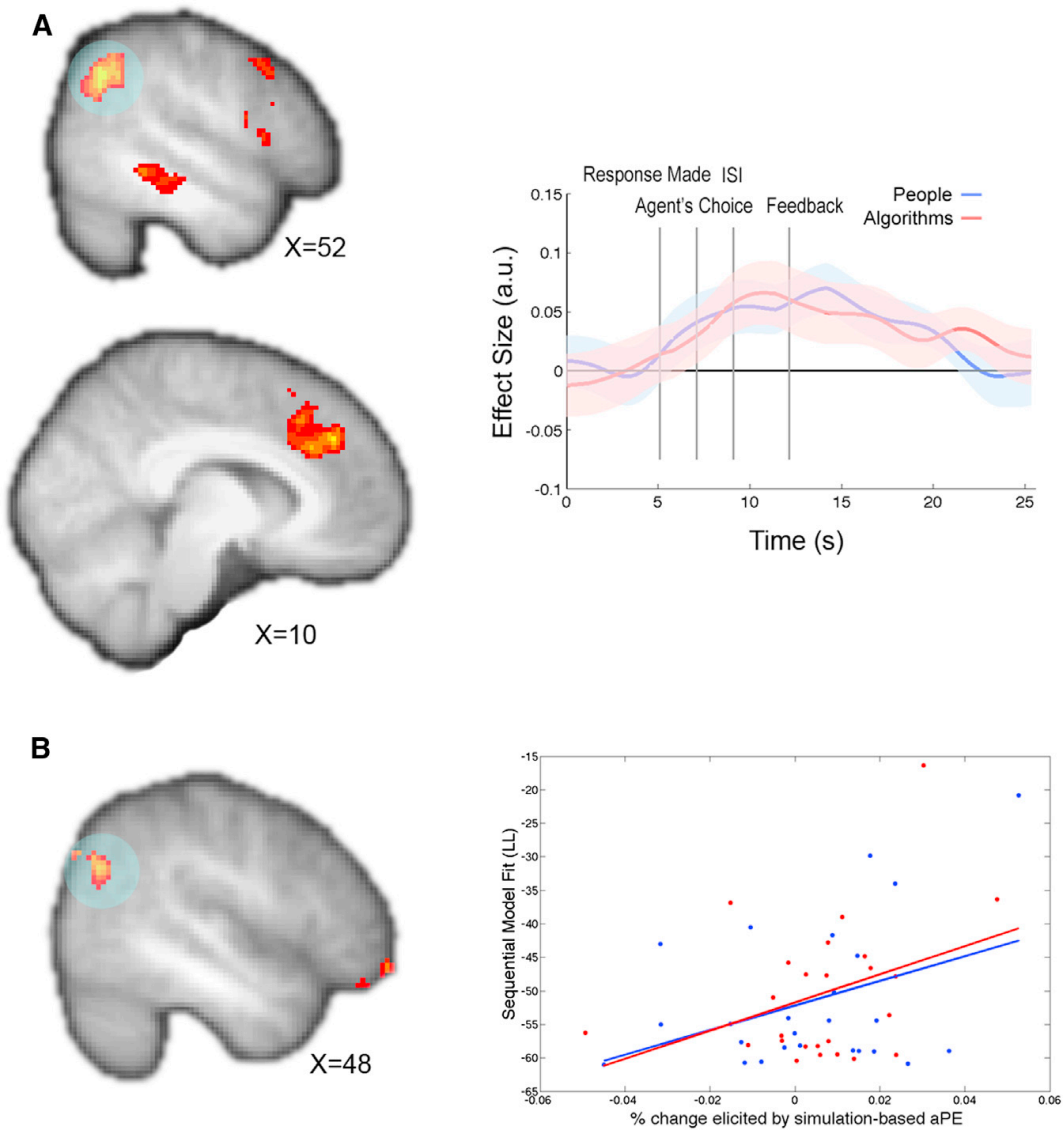
BOLD Effects of Simulation-Based aPEs (A) Left view shows Z-statistic maps (p = 0.05 cluster corrected) for the simulation-based aPE predicted by the sequential model, independent of agent type (people or algorithms), at the time of the observed agent’s choice. Right view shows the time course of the effect of this aPE in rTPJ (circled) plotted separately for people (blue) and algorithms (red) across the entire trial. Z-statistic map and time course are displayed according to the same conventions used in Figure 4. (B) Left view is a sagittal slice through Z-statistic map (p < 0.001 uncorrected for display purposes) relating to individual differences in the effect of simulation-based aPEs and the fit to behavior of the sequential model across people and algorithms. In the right view, a scatterplot of the percent signal change elicited by aPEs in independently identified rTPJ ROIs is plotted against the model fit for people (blue) and algorithms (red) separately.

The sequential model predicts that subjects will first update their beliefs about ability at the time they see the agent’s choice, based on whether or not it agrees with their own belief about the likely asset returns. Unsigned ability prediction errors (aPEs) time locked to this event revealed a network of brain regions frequently recruited during mentalizing tasks, including right temporoparietal junction (rTPJ), dmPFC, right superior temporal sulcus (rSTS)/middle temporal gyrus (rMTG), and an activation encompassing both ventral and dorsal premotor cortex (PMv and PMd, respectively) (Figure 5A; Z = 2.3, p = 0.05 corrected; Table S2). Independent time course analyses revealed largely overlapping effects of this simulation-based aPE when participants observed people and algorithms’ predictions (Figure 5A). Once again, we did not find any region that exhibited significantly different effects of simulation-based aPEs when subjects were observing people compared to algorithms.

To ascertain whether the neural representation of simulation-based aPEs in any brain regions might be behaviorally relevant, we tested whether individual differences in the choice variance explained by the sequential model were correlated with individual differences in the BOLD response to simulation-based aPEs. This whole-brain analysis revealed an overlapping region of rTPJ (Figure 5B; Table S3; p < 0.05 small volume corrected for a 725 voxel anatomical mask drawn around the rTPJ subregion identified by Mars et al., 2012). This analysis demonstrates that subjects whose behavior is better described by the sequential model have a stronger representation of simulation-based aPEs in rTPJ, suggesting that these learning signals are relevant to behavior.

### Evidence-Based Expertise Prediction Errors

**Figure 6:**
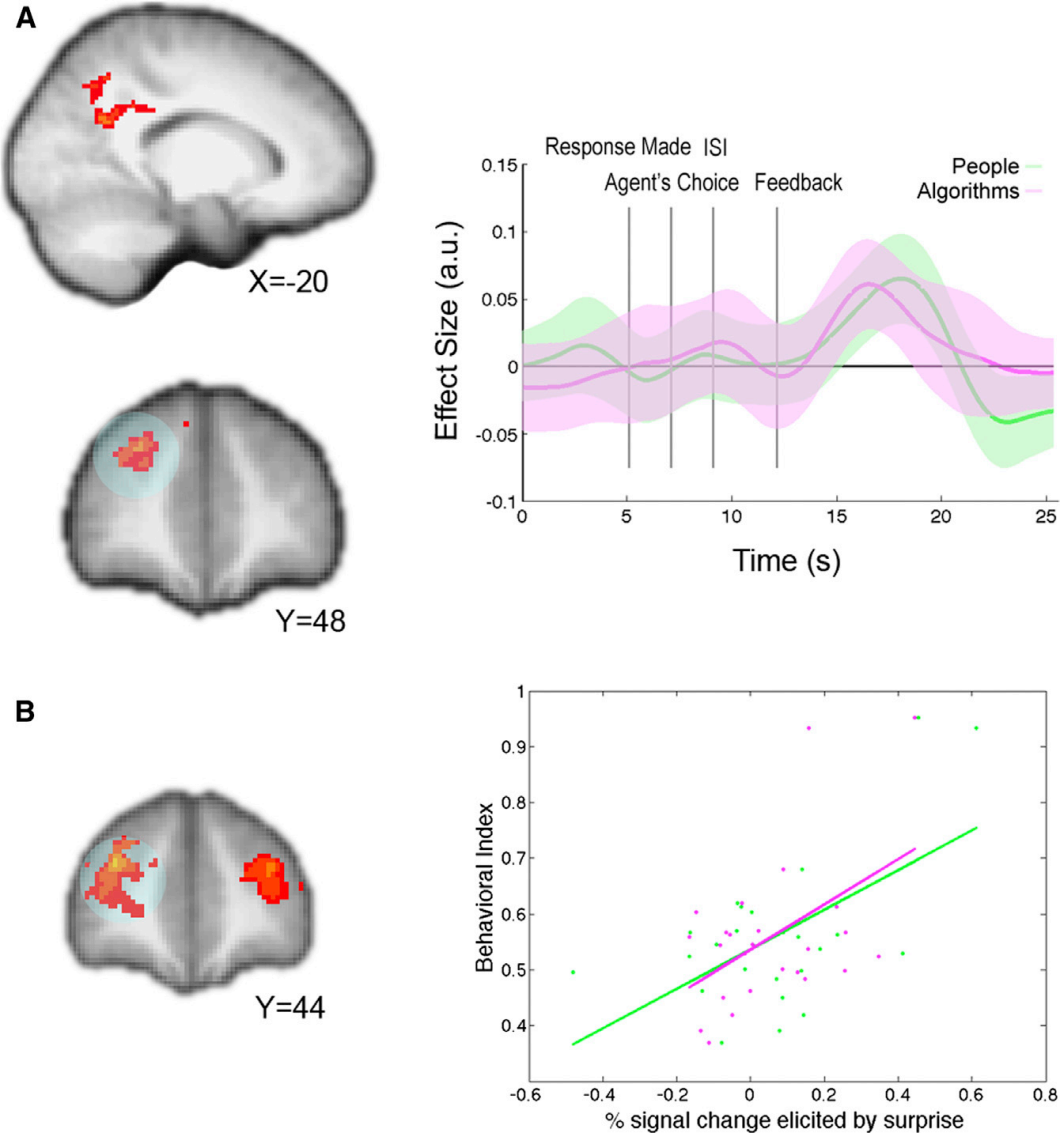
BOLD Effects of Evidence-Based aPEs (A) Left view shows Z-statistic maps (p = 0.05 cluster corrected) for the second aPE predicted by the sequential model, independent of agent type, at the time of feedback. In the right view, a time course of the effect in rdlPFC (circled) is plotted across the trial separately for people (green) and algorithms (magenta). Z-statistical map and time course are displayed according to the same conventions used in Figure 4. (B) Left view shows a Z-statistic map resulting from a between-subjects analysis of intersubject differences in relative behavioral fit (log likelihood) of the sequential and pure simulation models and the BOLD effect of evidence-based aPEs (p = 0.05 cluster corrected). In the right view, the percent signal change elicited by aPEs in independently identified rdlPFC ROIs (circled) is plotted against the relative model fit between sequential and simulation models (positive values indicate better fit of sequential compared to simulation model) for people (green) and algorithms (magenta) separately.

A third prediction made by the sequential model is a neural representation of a second aPE at the time subjects witness feedback indicating whether the agent’s choice was correct. Unsigned evidence-based aPEs time locked to this feedback event were significantly correlated with the BOLD response in right dorsolateral prefrontal cortex (rdlPFC) and lateral precuneus, independently of agent type (Figure 6A; Z = 2.3, p = 0.05 corrected; Table S2). Interrogation of the BOLD time course from independently identified rdlPFC ROIs on trials when subjects observed people and algorithms separately showed similar response profiles, both of which were time locked to feedback (Figure 6A).

Furthermore, similar rdlPFC and lateral precuneus regions showed greater neural responses to evidence-based aPEs in those individuals whose choices were better explained by the sequential model, as revealed by a whole-brain between-subjects analysis (Z = 2.3, p = 0.05 corrected; Table S3). This further shows that evidence-based aPEs are related to subjects’ behavior.

### Individual Differences in Learning and aPEs

We constructed a weighted semi-Bayesian variant of our sequential model to assess to what extent subject behavior was influenced by the evidence-based update as compared to the simulation-based update. This model included two additional free parameters, ρ and σ, that denote, respectively, the weight given to the simulation-based and evidence-based updates. See Supplemental Information for details. These parameters were estimated for each subject, and they effectively shift the distributions on ability up or down relative to the Bayesian sequential model (Figure S6). To compute a between-subject covariate that reflected the relative weighting of the evidence-based update, we normalized the relevant term by the sum of the two: σ/(ρ+σ). We found an overlapping region of rdlPFC that exhibited a strong relationship between this behavioral index and evidence-based aPEs (Figure 6B; Z = 2.3, p = 0.05 whole-brain corrected; Table S3). Moreover, analysis of independently identified ROIs revealed that this between-subject correlation was evident for both people (r = 0.58; p < 0.005) and algorithms (r = 0.48; p = 0.01). These analyses demonstrate that activity in the rdlPFC region correlates better with evidence-based aPEs in those individuals whose behavior is influenced more heavily by the evidence-based update than by the simulation-based update, further linking the neural signals and learning behavior.

### Neural Differences in Social Updating

**Figure 7:**
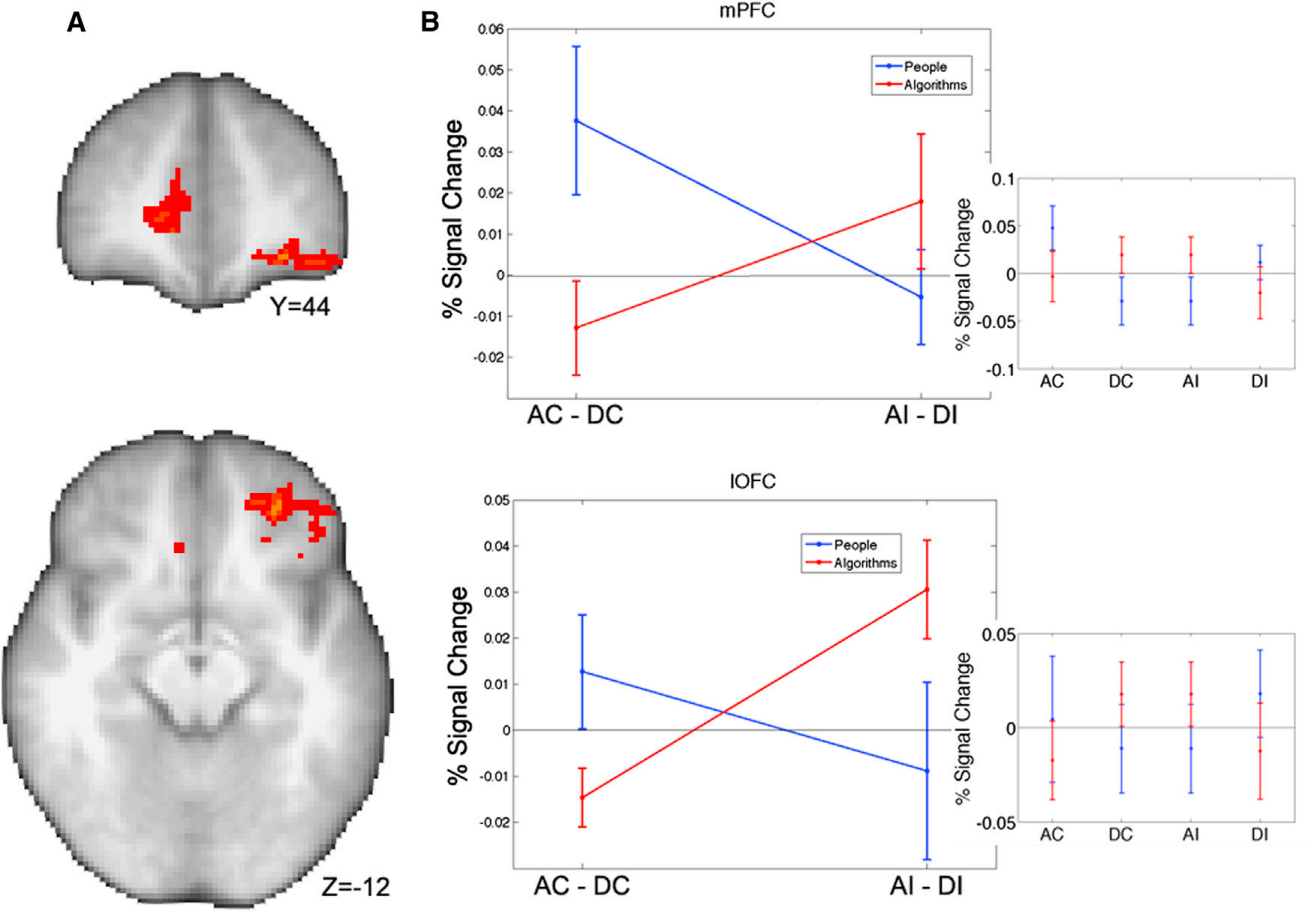
mPFC and lOFC Reflect Behavioral Differences in Ability Learning (A) Z-statistic maps (p = 0.05, cluster corrected) relating to the interaction between outcome type and agent type revealed in behavior (see Figure 2D) are shown. Z-statistic maps represent the following contrast between unsigned prediction errors at feedback: ((Agree and Correct − Disagree and Correct) − (Agree and Incorrect − Disagree and Incorrect)) × people − ((Agree and Correct − Disagree and Correct) − (Agree and Incorrect − Disagree and Incorrect)) × algorithms. Z-statistitcal map and time course are displayed according to the same conventions used in Figure 4. (B) Percent (%) signal change elicited by unsigned prediction errors for correct and incorrect agent predictions, when subjects would have likely agreed compared to disagreed, is plotted separately for people (blue) and algorithms (red). Plots on the far right show the same divided into the four outcome types separately: AC, DC, AI, and DI. See also Figure S4.

Agent performance can be attributed to ability or to chance. The behavioral regression analyses reported above show that subjects differentially credited specific agents for their correct and incorrect predictions in a manner that depended on the subjects’ own beliefs about the state of the asset. We investigated the neural processes associated with this effect, by searching across the whole brain for regions exhibiting significant effects of the following contrast between unsigned aPEs at feedback: ((AC−DC) − (AI−DI)) × people − ((AC−DC) − (AI−DI)) × algorithms. Significant whole-brain corrected clusters were found in left lOFC and mPFC only (Figure 7; Z = 2.3, p = 0.05, corrected; Table S3). Importantly, this analysis controls for differential updating between people and algorithms that is simply due to (1) correct versus incorrect predictions (because DC trials are subtracted from AC trials), and (2) predictions with which subjects would likely agree versus disagree (because AI−DI trials are subtracted from AC−DC trials). Moreover, there was a strong between-subject correlation between the behavioral interaction effect illustrated in Figure 2D and the neural interaction effect in independently defined lOFC ROIs (r = 0.55; p < 0.01).

To assess the robustness of the neural interaction effects in lOFC and mPFC, we repeated the analysis but replaced the regressors derived from the sequential model with ones derived from the model that allows for differential updating on AC, DC, AI, and DI trials for people and algorithms described above and in the Experimental Procedures. Unlike the sequential model, this model explicitly allows for the possibility of an interaction between agreement and correctness for people and algorithms. This analysis revealed very similar and overlapping effects in lOFC and mPFC for the same contrast between unsigned aPEs at feedback: ((AC−DC) − (AI−DI)) × people − ((AC−DC) − (AI−DI)) × algorithms (Figure S4B; Z > 3.1, p < 0.001 uncorrected).

## Discussion

One of the strongest determinants of social influence is the perceived ability or expertise of others (Aronson, 2003). Neurally, expert opinion has been shown to influence the valuation of obtained goods in ventral striatum, suggesting that it can modulate low-level reward processing (Campbell-Meiklejohn et al., 2010). Furthermore, prior advice has been shown to interact with learning from experience via an “outcome bonus” in the striatum and septum (Biele et al., 2011). Here, we investigated how beliefs about the expertise of others are represented and updated.

Computationally, we found that subjects used a model-based learning algorithm to learn the expertise of human and computer agents. Interestingly, the learning model was suboptimal for the task in two ways. First, subjects updated their expertise estimates both after observing the agent’s prediction (i.e., simulation-based updating) and after observing the correctness of the agent’s prediction (i.e., evidence-based updating). However, in the setting of the experiment, in which agents’ performance is determined by a constant probability of making a correct prediction independently of the state of the asset, only evidence-based updating is optimal. This may be because participants believed that agents were tracking the asset in a similar way to themselves, rather than performing at a constant probability. Second, subjects took into account their own beliefs about the asset when updating expertise beliefs, and they did this asymmetrically for human and algorithmic agents.

Neurally, we found that the key computations associated with the sequential model that best described behavior were reflected in brain regions previously implicated in aspects of social cognition (Behrens et al., 2009; Frith and Frith, 2012; Saxe, 2006), like the rTPJ, the aCCg, and rmPFC. The present study also extends the known roles of lOFC and mPFC in reward learning to updating beliefs about people and algorithms’ abilities. Furthermore, we found that reward expectations and rPEs were encoded in parallel in vmPFC and striatum, which are regions widely thought to be responsible for valuation, choice, and reward learning (Rangel and Hare, 2010; Behrens et al., 2009; Rushworth et al., 2011).

The computational model that best described subjects’ behavior predicts that they make their choices based on their belief about the agent’s expertise. Consistent with this prediction, responses in rmPFC, ACCg, and precuneus/PCC at the time of decisions were positively correlated with behavioral estimates about agents’ expertise. The model also predicts a simulation-based revision of expertise beliefs, just after subjects observe the agent’s choice. In line with this prediction, responses in rTPJ, dmPFC, rSTS/rMTG, and premotor cortex tracked unsigned simulation-based aPEs at that time. Finally, the sequential model predicts an evidence-based revision to subjects’ expertise estimates when they witness the final feedback. Accordingly, we found that responses in lateral precuneus and rdlPFC at this time increased with unsigned evidence-based aPEs. Together, these findings show localized neural activity for all of the key elements of the computational model.

The network found to encode expertise estimates during decisions has previously been implicated in component processes of social cognition. rmPFC has consistently been recruited in mentalizing tasks and has been suggested to play a top-down role in biasing information to be construed as socially relevant (Frith and Frith, 2012). Cross-species research has also suggested that ACCg plays a role in the attentional weighting of socially relevant information (Baumgartner et al., 2008; Behrens et al., 2008; Chang et al., 2013; Rudebeck et al., 2006), whereas activity in both the ACCg and posterior cingulate gyrus, which was also found to reflect expertise estimates, has been linked to agent-specific responses during the trust game (Tomlin et al., 2006). Here, we extend these findings by showing that these regions also play a role in representing another agent’s expertise when this information must be used to guide decision making. Furthermore, we show that intersubject variance in the fit of the sequential model explains variance in the neural fluctuations associated with tracking expertise in these same regions, and also in dmPFC.

Another set of brain regions, which includes rTPJ, dmPFC, and rSTS/rMTG, encoded simulation-based aPEs, when observing the agent’s choice. In order to compute simulation-based aPEs in our task, the subject must simulate his or her own prediction and then compare this with both the agent’s prediction and the agent’s estimated expertise level. The behavioral finding that learning depends on one’s own asset predictions and the neural identification of simulation-based aPEs complement recent demonstrations that simulation or modeling plays a central role in predicting others’ behavior (Nicolle et al., 2012; Suzuki et al., 2012). Activity in components of this network has repeatedly been reported during mentalizing (Frith and Frith, 2012; Saxe, 2006). Intriguingly, some of these regions are also consistently recruited during tasks that engage imagination, episodic memory, and spatial navigation (Buckner and Carroll, 2007; Mitchell, 2009), all of which may require some form of self-projection or simulation. The involvement of rTPJ, dmPFC, and STS/MTG in updating estimates about others’ expertise through simulating their own prediction accords with previous demonstrations that these regions encode prediction errors in situations where subjects simulate either the intentions of a social partner (Behrens et al., 2008) or the likely future behavior of a confederate (Hampton et al., 2008). Recent studies have examined the relative contributions of structures in the mentalizing network to aspects of social cognition (e.g., Carter et al., 2012). In our study, we did not find any clear differences between these regions in tracking expertise, although multivariate approaches may prove more sensitive to any such differences.

Activity in yet another pair of brain regions, rdlPFC and lateral precuneus, reflected aPEs when subjects revised expectations at feedback, and in parallel to rPEs identified in striatum. Similar regions have been implicated in executive control and, intriguingly, have recently been shown to encode model-based state prediction errors (Gläscher et al., 2010). Moreover, activity in rdlPFC elicited by evidence-based aPEs reflected individual differences in subjects’ relative reliance on evidence-based aPEs, compared to simulation-based aPEs, during learning. Activity in this region therefore reflects individual differences in the extent to which learning is driven by correct agent performance or subjects’ own beliefs about the best prediction.

We found that subjects credited people more than algorithms for correct predictions that they agreed with rather than with correct predictions that they disagreed with. In fact, subjects gave substantial credit to people for correct predictions they agreed with but hardly gave them any credit for correct predictions they disagreed with, whereas this distinction had little impact on crediting algorithms for correct predictions (see Figure 2D). Furthermore, subjects penalized people less than algorithms for incorrect predictions with which they agreed compared to disagreed. This difference in learning about people and algorithms is striking because the only difference between them in our study was the image to which they were assigned. A key open question concerns what factors control the construction of the prior categories that lead to this behavioral difference. We speculate that one source of the difference between people and algorithms may be related to the perceived similarity of the agent to the subject. It is likely that subjects thought of the human agents as more similar to themselves, which may have led them to relate or sympathize more with people than with algorithms as a function of their own beliefs about what constituted a reasonable choice.

This differential updating for people and algorithms was reflected in brain regions thought to be important for contingent learning in nonsocial contexts (Tanaka et al., 2008; Walton et al., 2010), suggesting that social and nonsocial contingent learning share neuroanatomical substrates. Interestingly, there was a tendency for the neural interaction effects to be driven by people in mPFC, a region also linked to social cognition, and algorithms in lOFC, although the difference was not significant. We did not identify any brain regions that were specific to learning about the expertise of people or algorithms in our study. Rather, lOFC and mPFC appear to be utilized differentially in ways that corresponded to behavioral differences in learning about people and algorithms.

Many of our analyses revealed common recruitment of regions often associated with mentalizing when subjects used or revised beliefs about people and algorithms. Notably, most other studies investigating the computations underlying social learning have not incorporated matched human and nonhuman controls (Behrens et al., 2008; Cooper et al., 2010; Hampton et al., 2008; Yoshida et al., 2010). It may also be important that our algorithm possessed agency in that they made explicit predictions, just as people did. It is therefore possible that some of the neural computations underlying social learning about humans and nonhuman agents are alike because they both recruit the same underlying mechanisms. This interpretation is consistent with a recent demonstration that dmPFC activity tracks the entropy of a computer agent’s inferred strategy during the “stag hunt” game (Yoshida et al., 2010). It is also possible that learning about expertise is distinct from learning about intentions, dispositions, or status (e.g., Kumaran et al., 2012), which people might be more likely to attribute to humans than to nonhuman agents.

One important methodological aspect of the study is worth highlighting. Behaviorally, we find evidence in support of a Bayesian model of learning, in which subjects update their ability estimates whenever they observe useful information. Importantly, we also find evidence that neural activity in the networks described above covaried with unsigned prediction errors at the time of these two updates. Because prediction error activity is more commonly associated with non-Bayesian reinforcement-learning algorithms than with Bayesian learning, we provide some elaboration. Notably, in our study, unsigned prediction errors at choice and feedback were indistinguishable from the surprise about the agent’s prediction or outcome (−p(log_2_(p(g_t_)); mean correlation, r = 0.98). One possibility is that the unsigned aPEs reflect the amount of belief updating that is being carried out in these areas, rather than the direction of updating (see Supplemental Experimental Procedures and Figure S7 for a direct comparison between aPEs and Bayesian updates). In particular, unsigned aPEs are high when subjects’ mean beliefs about the agents’ abilities are close to 0.5, at which point information about agents’ bets or accuracy generally induces substantial updating in our task. On a neuronal level, these may reflect (1) content-selective attentional weighting or surprise signals (see Roesch et al., 2012 for a discussion of such signals in reinforcement learning); (2) within- and/or between-subject variation in the direction of signed aPEs; or (3) spatial intermixing of signed and unsigned aPE neurons at a spatial scale that cannot be resolved with fMRI. We also emphasize that the objective of this study is not to make a strong claim about whether or not computations about expertise necessarily involve a Bayesian updating mechanism. Rather, the Bayesian algorithms used here provide a tractable framework through which we have been able to implicate specific neural structures in mediating computations important for tracking expertise.

Although it is unlikely that subjects uncovered the full structure of the process underlying the agents’ predictions, it is nonetheless the case that the agents in our task did not learn to track the asset behavior (because their performance stayed constant throughout the study). We therefore use the term “expertise” loosely to refer to the participants’ beliefs about the performance level of an agent within a specified domain. This is most likely to be an oversimplification in the real world, where an agent’s expertise is likely to depend on context. For example, someone might be good at picking winning stocks in bull markets, but not in bear markets; or might be good at forecasting stocks, but not bonds. Furthermore, the difficulty of the setting will modulate real-world agent performance and likely expertise judgments. Determining the role of these contextual factors in evaluating others will provide a richer characterization of social learning in naturalistic settings.

## Experimental Procedures

### Subjects

A total of 31 human subjects participated in the experiment. Two subjects were removed from further analysis due to excessive head motion, one because of experimenter error during data collection, and three because they showed no behavioral evidence of learning, resulting in 25 subjects (eight females/17 males, mean age 25 years, age range 18–30). We excluded volunteers who were not fluent English speakers and who had any history of a psychiatric or neurological disorder. All subjects provided informed consent prior to their participation following the rules of Caltech’s IRB.

### Task

Subjects performed a task in which they had to learn about the performance of a financial asset, as well as about the ability of human and computerized agents who would predict the performance of the asset. Every trial, the asset went up with probability *p*TRUE_*t*_ and down with probability *1-p*TRUE_*t*_. These probabilities evolved over the course of the trial according to the time series shown in Figure 2B (dashed line). Each element of *p*TRUE_*t*_ was drawn independently from a beta distribution with a fixed variance (SD, 0.07) and a mean that was determined by the true reward probability on the preceding trial. This functional form was selected, together with all other parameters of the task, to reduce the correlation among the fMRI parametric regressors described below (e.g., see Figure S5).

Subjects made decisions in three types of trials (see Figure 1). In condition 1, they were presented with a face picture of a human agent and had to decide whether to bet for or against the agent. After a brief delay, they observed the agent’s prediction about the asset performance (up/down). Following a jittered interstimulus interval, feedback was presented indicating whether the asset’s value went up or down, as well as feedback for the trial. The subject made $1 if she guessed correctly the performance of the agent (i.e., if she bet for him, and he was correct, or if she bet against him, and he was mistaken) and lost $1 otherwise. This screen also indicated the performance of the asset with an up/down arrow, independently of any other contingencies for the trial. The feedback phase was followed by a jittered intertrial interval.

Condition 2 was identical to condition 1, except that now the agent was depicted by a 2D fractal image and described to the subjects as a computerized-choice algorithm. In contrast, in condition 1, the agent was described as depicting the predictions of a real person that had made predictions in a prior testing session. This was indeed the procedure implemented, although the choices that the real person made in the prior testing session were predetermined by choices generated by the probabilities shown in Figure 2A.

In condition 3, there was no agent and thus no ability prediction. Instead, the subject had to predict whether the asset would go up or down. The participant’s payoff in this case depended on the ability to predict the next outcome of the asset correctly: $1 for correct guesses, and −$1 for incorrect ones.

We emphasize that in all of the conditions, the subject’s payoff depended on the quality of his guesses, and not on the actual performance of the asset or of the agents. At the end of the experiment, subjects were paid their total earnings in cash.

The task was divided into four fMRI blocks (or runs) of 55 trials. In each block, the subject observed the predictions of three agents (either two people and one algorithm, or the reverse). There were 11 asset prediction trials per block. Subjects made predictions about each of the three agents in a block in an equal or nearly equal number of trials (14 or 15 trials each, depending on the block). The three agents and asset prediction trials were randomly interleaved with the constraint that the same stimulus (agent or asset) was never repeated. In total, this allowed for 88 trials observing people, 88 trials observing algorithms, and 44 asset prediction trials.

There were four people and four algorithms in total. Each agent was characterized by a fixed ability α denoting the constant and independent probability with which he made the correct prediction for the asset’s performance in every trial. Note that the agents with correct performance 0.6 and 0.4 repeated in a later block but that those with 0.3 and 0.7 performance did not (see Figure 1B). Because the estimated probabilities for asset price increases fluctuated primarily between 0.25 and 0.75 (see Figure 2B), agent performance seldom reached unreasonably high or low levels given the predictability of the asset.

Figure 1B summarizes the agent configuration and parameters used in the experiment. For the human agents, we used male faces of the same approximate age to minimize any potential inferences of ability based on age or gender-related cues. Assignment of specific faces and fractal images to agent predictions was pseudorandomly determined and counterbalanced across subjects.

Importantly, at the beginning of the experiment, subjects were told that the asset performance evolved over time but were not given the details of the specific process. In addition, they were told that real people and computerized algorithms programmed by the experimenters to track the asset had previously made predictions about whether the asset would increase or decrease in value and that those constituted the predictions that they would bet on. They were also informed that the identities of the faces displayed did not correspond to the actual people who had made the prior predictions. Finally, they were told that people agents were selected such that they differed in their abilities to track the asset, and likewise for algorithms.

### Behavioral Models

We compared the extent to which various models could account for the subjects’ behavior when predicting the agent’s ability and the performance of the assets. Except for the Full Model, these models consisted of two separable components: a model for the performance of the asset, and a model of the agent’s ability. These models use the history of observed evidence to update beliefs about the agents’ abilities and about the state of the asset.

### Asset Learning Model

The model of how subjects learn the probability of asset price changes is based on previous work on Bayesian reward learning (Behrens et al., 2007, 2008; Boorman et al., 2011). A detailed description of this model and its estimation is provided in the Supplemental Information, as well as in the supplemental tables and figures of these studies; for example, Behrens et al. (2007).

### Bayesian Learning about an Agent’s Expertise

We considered four distinct but natural classes of behavioral models. We refer to the classes as the full model, pure evidence model, the pure simulation model, and the sequential model. A formal description of the full model is provided in the Supplemental Information. Let *q*_*t*_ denote the probability that the asset goes up at time t, according to the subject’s beliefs at the time.

The remaining models have some common properties, which we discuss first. Inferences about agent expertise are made based on the performance of the agent’s guesses. Let *g*_*t*_ denote the subject’s belief about the quality of the guess made by the agent presented at time t. In a slight abuse of notation, let *g*_*1:t*_ denote the quality history of the agent’s guesses, with *1* indexing the first time the agent was active, 2 the second time it was active, etc. At every active time step *t*, *g*_*t*_ = 1 if the agent’s choice is judged to be of good quality, and *g*_*t*_ = 0 otherwise. The subject assumes that the agent’s ability is described by the constant but unknown parameter α describing the agent’s (independent) probability of making the right guess in every trial. In all of the models, subjects update their beliefs about α using optimal Bayesian inference. Under these assumptions, if the model starts the learning process with uniform priors over all ability levels, the posterior beliefs are known to have a very simple form (Jackman, 2009):

$$p\left({\text{α}}_{t+1}|g_{\mathrm{1:t}}\right)=\text{Beta}\left(s\left(g_{\mathrm{1:t}}\right),f\left(g_{\mathrm{1:t}}\right)\right),$$

where

$$s\left(g_{\mathrm{1:t}}\right)=1+\text{\# correct}\;\text{guesses}\;\text{in}\;g_{\mathrm{1:t}}$$

and

$$f\left(g_{\mathrm{1:t}}\right)=1+\text{\# incorrect}\;\text{guesses}\;\text{in}\;g_{\mathrm{1:t}}.$$

Let (α_*t+1*_) denote the mean ability level in the posterior distribution, and let *b*_*1:t*_ denote the subject’s history of bets in any trial *t* involving an agent (i.e., in conditions 1 or 2). All of the models assume that subjects chose their bet according to the following soft-max distribution:

$$P\left(b_{t}=for\right)=\frac{1}{1+\exp\left(-\beta \left(mean\left(\alpha_{t}\right)-0.5\right)\right)}$$

where β is a subject-specific free parameter that reflects the sensitivity of subjects’ bets to their expertise estimates. *P*(*b*_*t*_ *= against*) *=* 1 − *P*(*b*_*t*_ *= for*).

The models differ from each other in the information that they use to judge the agents’ guesses as correct or incorrect and on when the ability beliefs are updated.

According to the *pure evidence model*, subjects judge the performance of the agents based only on the correctness (*c*_t_) of their guesses at the end of the trial. Note that *c*_t_ = 1 if the agent guesses the performance of the asset in trial *t* correctly, and *c*_t_ = 0 otherwise. Because *g*_*t*_ denotes the subject’s judgment about the quality of the agent’s action, in this model, we have that *g*_*t*_ *=* 1 if *c*_t_ = 1, and *g*_*t*_ *=* 0 otherwise (i.e., if *c*_t_ = 0). Because the correctness information is only revealed at the end of the trial, in this model, beliefs are only updated at that time. Note that because agent performance was in fact independent from the asset value, the evidence model is the best updating strategy given the true parameters of the task.

In contrast, in the *pure simulation model*, subjects judge the performance of the agents based on whether or not they conform to their own beliefs about the asset. Thus, in this case, *g*_*t*_ *=* 1 if the agent chooses up (*a*_*t*_ = 1) when the subject also believes that the asset is likely to go up (*q*_*t*_ > 0.5) and chooses down (*a*_*t*_ = 0) when the subject believes that the asset is likely to go down (*q*_*t*_ < 0.5), and *g*_*t*_ *=* 0 otherwise. Because this information is revealed at the time of the agents’ choices, in this case, expertise beliefs are updated in the middle of the trial.

Finally, the *sequential model* combines the two updates, which are carried out sequentially. In particular, it predicts that subjects update their beliefs twice: first upon observing how the agent’s choice compares to their own beliefs about the likely asset performance, and second, at the end of the trial based on the correctness of the agent’s prediction. Let *u* denote the temporal order of the update within a trial (i.e., *u* = 1 for the first update and *u* = 2 for the second update). In this case, the judgment at the time the agent’s prediction is observed is given by

$$g_{t}^{u}=1\;\text{if}\;\left(a_{\text{t}}=1\;\text{and}\;q_{\text{t}}>0.5\right)\;\text{OR}\;\left(a_{\text{t}}=0\;\text{and}\;q_{\text{t}}<0.5\right)$$

$$g_{t}^{u}=0\;\text{otherwise},$$

and the judgment at the end of the trial is given by

$$g_{t}^{u}=1\;\text{if}\;c_{\text{t}}=1$$

$$g_{t}^{u}=0\;\text{otherwise}.$$

The ability belief updated at each time step is the most recent estimate. We also considered several reinforcement-learning (non-Bayesian) versions of these three models, none of which performed as well as their Bayesian counterparts (see Supplemental Information for details).

### fMRI Data Analysis

fMRI analysis was also carried out using FSL (Jenkinson et al., 2012). A GLM was fit in prewhitened data space. A total of 28 regressors (and their temporal derivatives, except for the 6 motion regressors produced during realignment) were included in the GLM, one for each of the four runs/sessions collected during scanning: the main effect of the first decision making phase for predictions about people (condition 1), algorithms (condition 2), and assets (condition 3); the main effect of the observed agent’s prediction for people (condition 1) and algorithms (condition 2); the main effect of the interstimulus interval (conditions 1 and 2); the main effect of the feedback phase for AC, DC, AI, and DI trials for people (condition 1) and algorithms (condition 2); the main effect of the feedback phase for assets (condition 3); the main effect of the presentation screen at the beginning of each run; the interaction between chosen subjective EV and the decision making phase separately for people, algorithms, and assets; the interaction between expertise and the decision making phase separately for people and algorithms; the interaction between simulation-based aPEs and the other agent’s prediction separately for people and algorithms; the interaction between rPE and feedback phase separately for people, algorithms, and assets; the interaction between evidence-based aPEs and feedback phase separately for AC, DC, AI, and DI trials separately for people and algorithms; and 6 motion regressors. The ITI event was not modeled. See the main text for the definition of the AC, DC, AI, and DI trials.

We defined additional contrasts of parameter estimates (COPEs) for expertise and expertise prediction errors of agents, independent of agent type, as a (1 1) contrast of relevant regressors based on the people and algorithms, as well as COPEs for the difference (1 −1) between expertise and expertise prediction errors for people compared to algorithms. To search for common expertise prediction errors at feedback, we defined a ((AC + DC) + (AI + DI)) × people + ((AC + DC) + (AI + DI)) × algorithms) contrast. To search for differences between people and algorithms that depended on an interaction between agreement and correctness, as was revealed in behavior, we defined the following difference contrast: ((AC − DC) − (AI − DI)) × people − ((AC − DC) − (A I− DI)) × algorithms. Similarly, we defined COPEs for chosen subjective EV and rPE as a (1 1 1) contrast of relevant regressors based on people, algorithms, and assets. Aside from the motion regressors, all regressors were convolved with FSL’s default hemodynamic response function (gamma function, delay is 6 s, SD is 3 s) and filtered by the same high-pass filter as the data. COPEs were combined across runs using a fixed effects analysis. See Supplemental Information for more details of fMRI acquisition, preprocessing, and analyses.

## Figures and Tables

**Table 1:** Model Comparison

Model	Params (per Subject)	λ_(p)_ (Mean per Subject)	λ_(a)_ (Mean)	β (Mean)	λ_(s)_ (Mean)	NlogL (Sum)	BIC (Sum)
Sequential Bayes	1	NA	NA	4.45	NA	3,018.8	6,253.0
RL sequential 2P	2	0.06	NA	6.78	NA	3,007.7	6,446.0
Bayes evidence	1	NA	NA	3.52	NA	3,158.0	6,531.3
RL sequential 3P	3	0.06	0.06	7.18	NA	3,058.7	6,763.3
Bayes simulation	1	NA	NA	2.79	NA	3,286.8	6,789.0
RL evidence 2P	2	0.05	NA	6.84	NA	3,245.7	6,922.1
RL simulation 2P	2	0.045	NA	6.47	NA	3,275.4	6,981.4
RL evidence 3P	3	0.064	0.063	6.97	NA	3,194.5	7,034.8
Full model	1	NA	NA	6.56	NA	3,414.1	7,043.6
RL simulation 3P	3	0.054	0.045	7.22	NA	3,254.3	7,154.5
RL evidence 4P	4	0.095	0.087	4.51	0.20	3,261.6	7,384.5

A comparison of several alternative models is presented, including the number of parameters (Params) in the model (per subject), the mean value for terms in the models (where applicable), the negative log likelihoods (NlogL; summed [Sum] over participants), and the BIC (summed over participants). Lower values indicate better fits to behavior. λ_(p)_, learning rate for people; λ_(a)_, the learning rate for algorithms (where applicable; if there is only one learning rate, then this is denoted by λ_(p)_); λ_(s)_, the learning rate for asset tracking; β, the inverse temperature (choice-sensitivity parameter).

## Supplemental Information

Document S1. Supplemental Experimental Procedures, Figures S1–S7, and Tables S1–S3
