# Supplementary material for: The Behavioral and Neural Mechanisms Underlying the Tracking of Expertise
Source: Neuron. 2013 Dec 18;80(6):1558–71. doi: 10.1016/j.neuron.2013.10.024 (PMC3878380; doi:10.1016/j.neuron.2013.10.024)
Supplement: Document S1. Supplemental Experimental Procedures, Figures S1–S7, and Tables S1–S3 [file mmc1.pdf]

Neuron, Volume *80*

Supplemental Information

The Behavioral and Neural Mechanisms Underlying the Tracking of Expertise

Erie D. Boorman, John P. O'Doherty, Ralph Adolphs, and Antonio Rangel

SUPPLEMENTAL INFORMATION

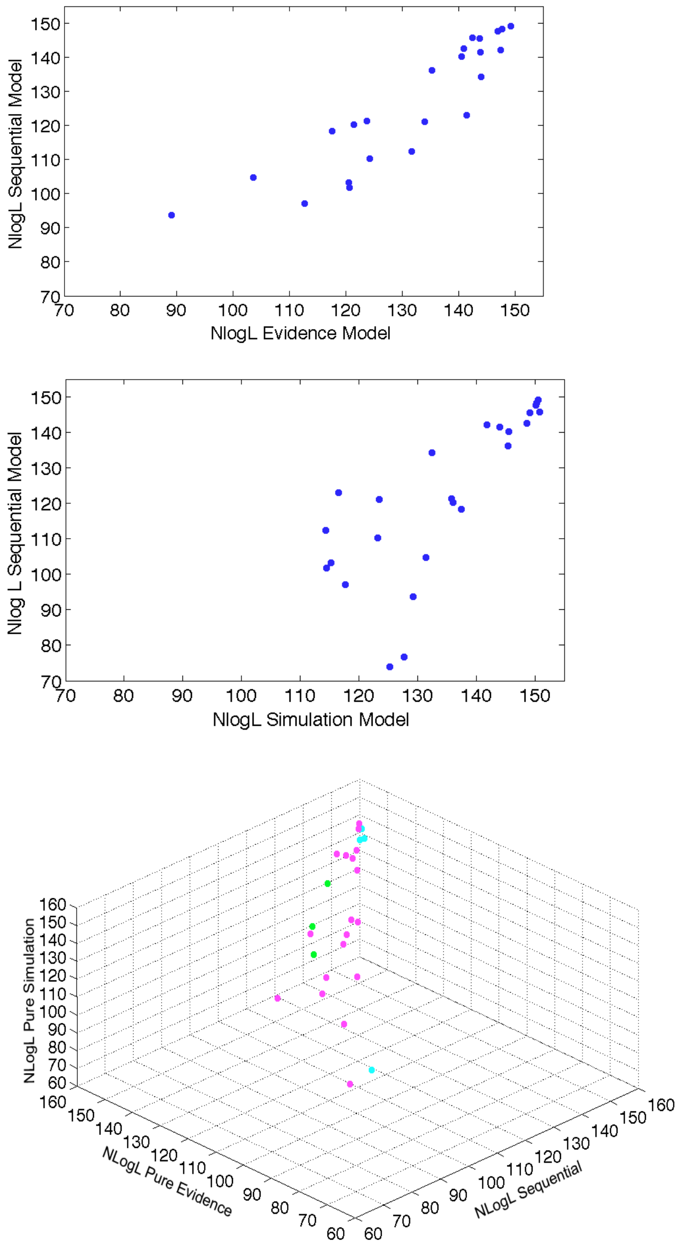

Figure S1. Fit of main behavioral models. Top: Scatter plot shows the fit of the sequential Bayesian model, against the evidence Bayesian model. Middle: the same is shown against the simulation Bayesian model. Superior fit of the sequential model can be seen in most subjects, as they lie beneath the diagonal. Bottom**:** 3D scatter plot shows the fit (NLogL: negative log likelihood) of the sequential model, the pure evidence model, and pure simulation model in our sample of participants. Each circle represents a single subject. Subjects whose behavior is better described by the sequential model, the evidence model and the simulation model are colored in magenta, cyan, and green respectively. Lower values indicate better fits.

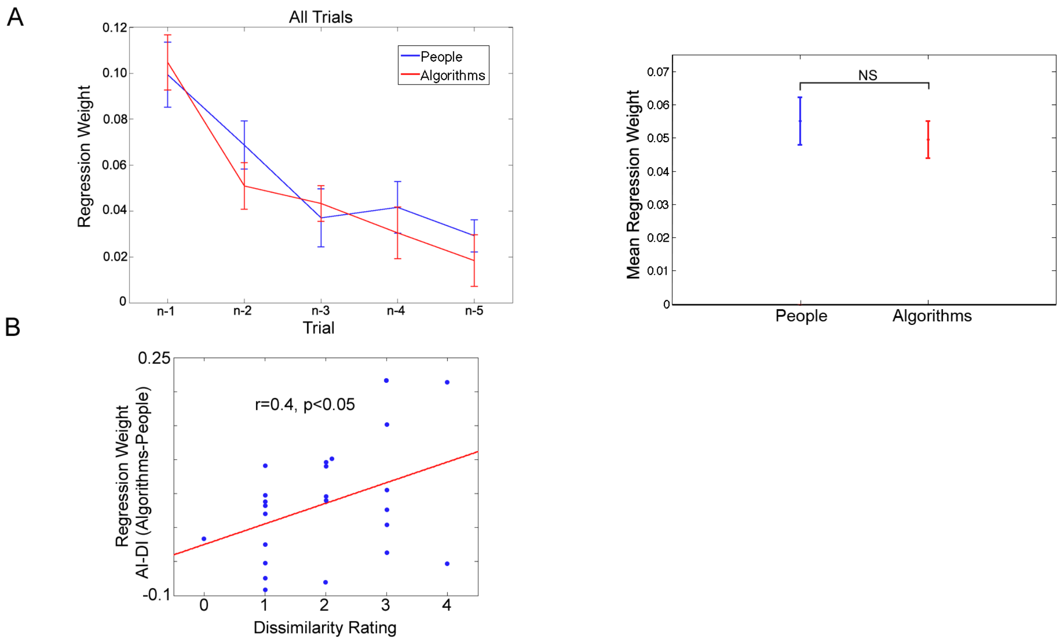

Figure S2. Behavioral regression results, related to Figure 2D. (A) Left: Regression coefficients relating to the influence of previous outcomes (correct and incorrect agent predictions for all trials) on subjects' current choices (bets for or against that particular agent) for people (blue) and algorithms (red). As predicted the regression weights decrease with increasing delay (trials n-1 to n-5). Right: The mean of these coefficients pooling all trials reveals no difference between people and algorithms. (B) Scatterplot illustrating the relationship between an individual's post-scan dissimilarity rating (between people and algorithms) and the difference between regression weights on incorrect trials when subjects would agree compared to disagree for algorithms compared to people.

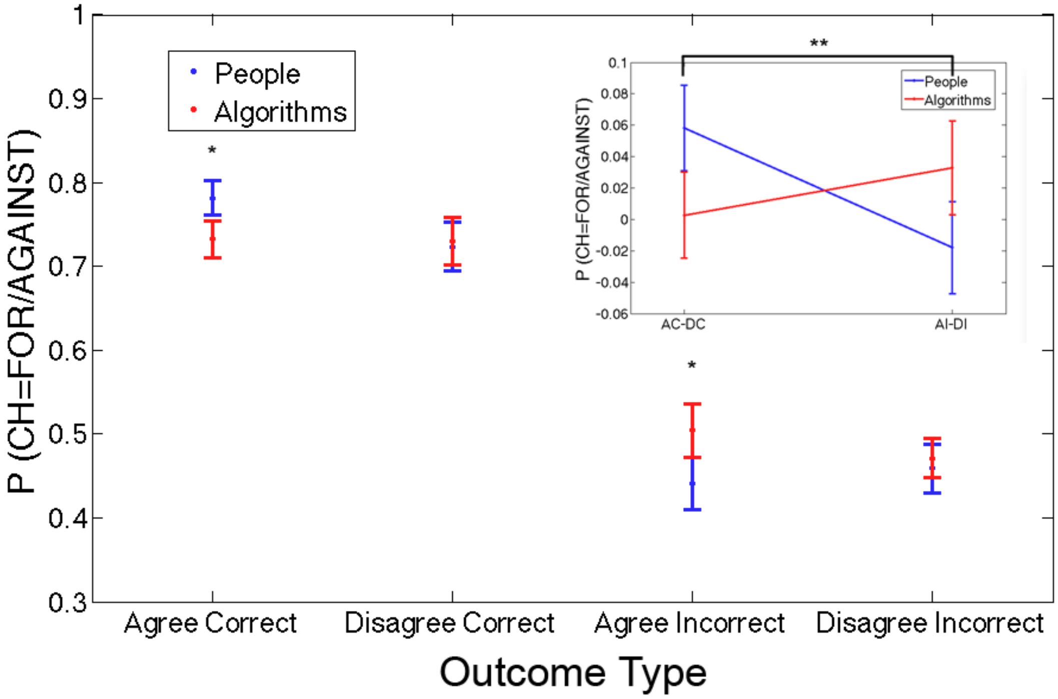

Figure S3. Choice frequency analysis. Mean frequency of betting for (P(Choice = For)) an agent on correct trials and betting against (P(choice = AGAINST)) an agent on incorrect trials is plotted following different previous trial outcomes for that particular agent (n-1). Frequencies are shown following Agree and Correct trials (AC), Disagree and Correct trials (DC), Agree and Incorrect trials (AI), and Disagree and Incorrect trials (DI). Circles represent means and error bars represent SEM. Inset: The same is plotted with DC trials subtracted from AC trials and DI trials subtracted from AI trials. * denotes p<0.05, ** p<0.01.

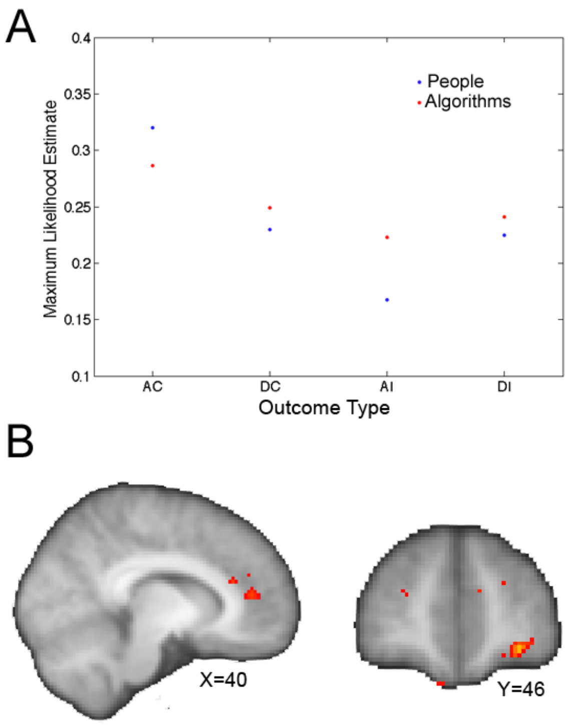

Figure S4. Fixed effects results. (A) Maximum likelihood estimates for learning rate parameters on AC, DC, AI, and DI outcome types for the differential model from a fixed effects group analysis are plotted for people and algorithms separately. Largest differences between peole and algorithms occur on AC and AI trials. (B) Z-stat maps showing effects in mPFC and lOFC from the following contrast between unsigned prediction errors derived from the differential model at feedback [(Agree and Correct – Disagree and Correct) – (Agree and Incorrect – Disagree and Incorrect)]PEOPLE – [(Agree and Correct – Disagree and Correct) – (Agree and Incorrect – Disagree and Incorrect)]ALGORITHMS. Maps are thresholded at Z>3.1, p<0.001 uncorrected.

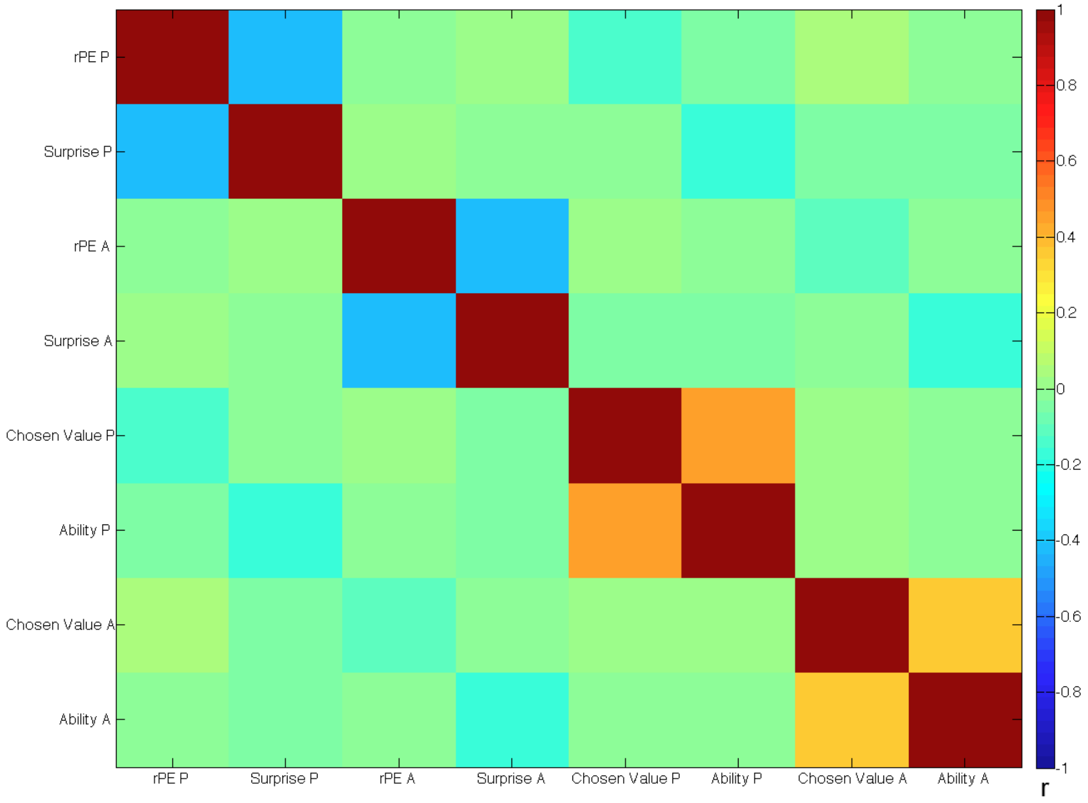

Figure S5. Correlation matrix of task variables. Figure depicts the Pearson correlation coefficient for regressors from conditions 1 and 2 alone. Specifically, shown are reward prediction error at feedback when subjects observe people (rPE P), the unsigned ability prediction error at feedback for people (Surprise P), reward prediction error at feedback when subjects observe algorithms (rPE A), the unsigned ability prediction error at feedback for algorithms (Surprise A), chosen expected value at decision when subjects observe people (Chosen Value P), the ability estimate for people (Ability P), chosen expected value at decision when subjects observe algorithms (Chosen Value A), the ability estimate for algorithms (Ability A).

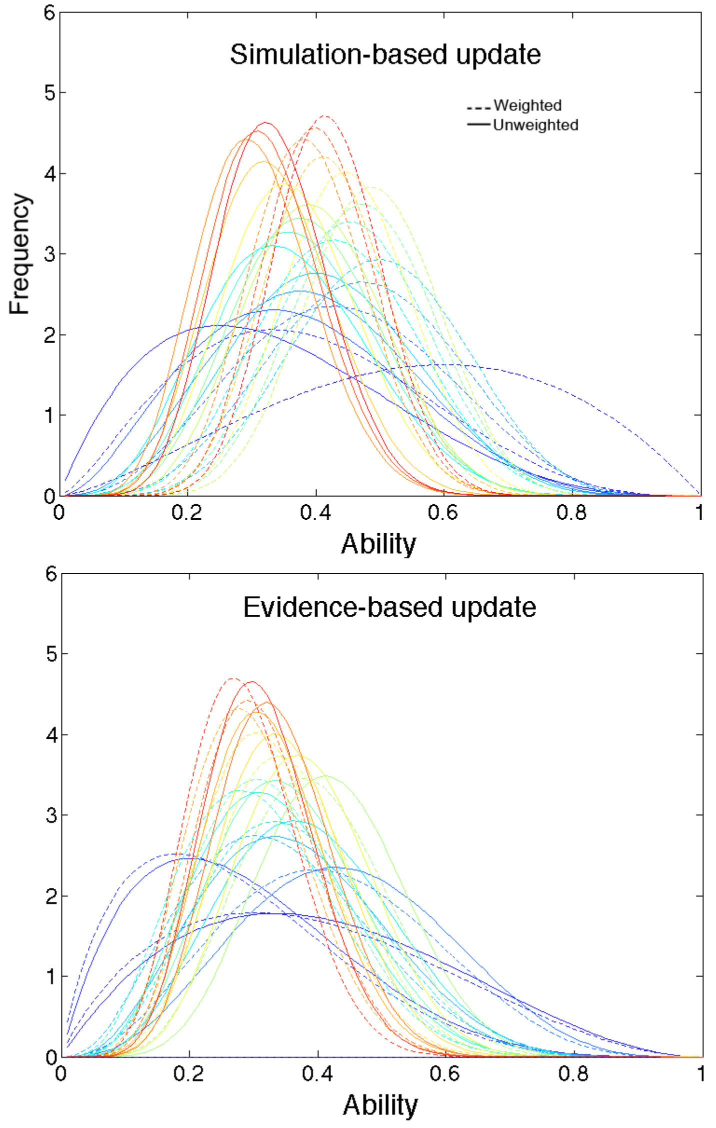

Figure S6. Weighted and non-weighted sequential model distributions. Posterior belief distributions over ability level following a simulation-based update (top) and an evidence-based update (bottom) for one example subject, who had MLEs of ρ=1.49 and σ=0.87 for the weighted sequential model. These parameters effectively shift the distributions on ability up or down relative to the Bayesian sequential model. The solid lines are from the sequential Bayesian model and the dashed lines are from the semi-Bayesian weighted sequential model.

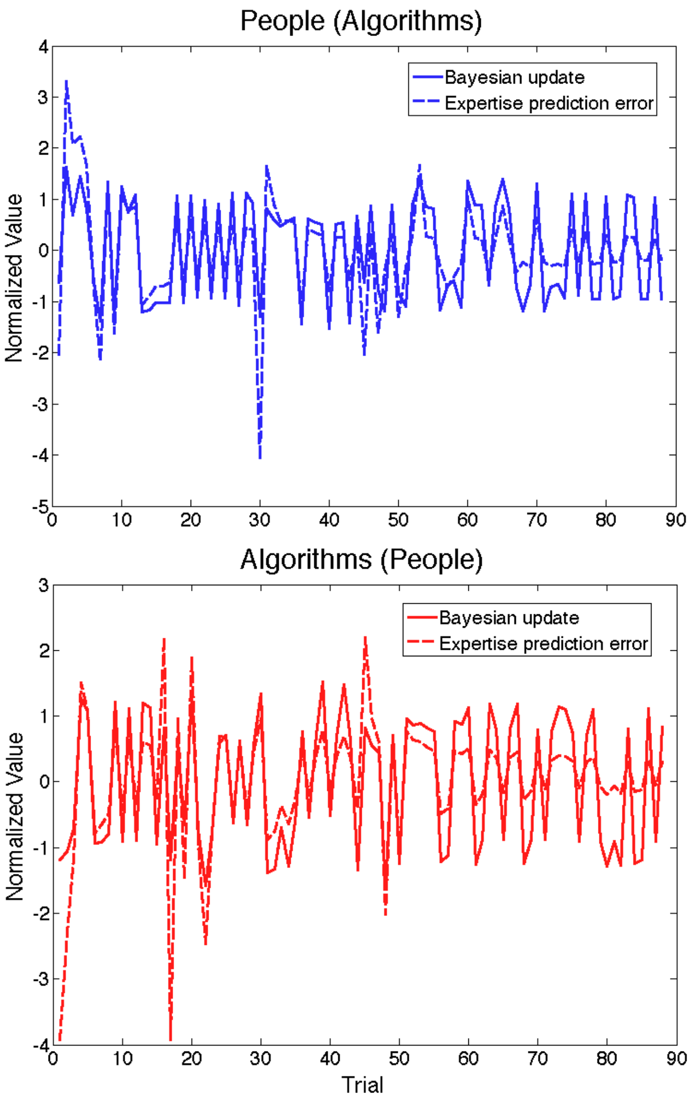

Figure S7. Relationship between evidence-based aPEs and Bayesian updates. Signed Bayesian updates and signed ability prediction errors at feedback for the sequence of observed people and algorithm agents are shown in blue and red respectively. Parentheses in the titles indicate that assignment to people or algorithms was counterbalanced across participants. As can be seen, these terms are highly correlated in both cases (mean r = 0.74). Notably, the Bayesian update is generally slightly larger early during learning about a new agent and slightly smaller following many observations with that agent. These two related variables were too highly correlated to differentiate in the current study.

Model	Parameters (per subject)	β (mean per subject)	$\lambda_{(s)}$ (mean per subject)	NlogL (sum)	BIC (sum)
Bayes	1	6.73	NA	516.4	1207.9
RL	2	3.75	0.22	555.3	1460.8

Table S1. Model comparison of asset tracking. A comparison of Bayesian and reinforcement-learning asset tracking models, including the number of parameters in the model (per subject), the mean value for terms in the models (where applicable), the negative log likelihoods (summed over participants), and the Bayesian Information Criterion (summed over participants). $\boldsymbol{\lambda_{(s)}}$ denotes the learning rate for asset tracking; **β** denotes the choice sensitivity parameter; NlogL denotes negative log likelihood; BIC denotes Bayesian information criterion. Lower values indicate better fits to behavior.

Contrast	Region	Peak Coordinates (MNI)	Z - Max	Extent
Chosen Value at decision	vmPFC*	8, 38, -14	3.80	13
	Planum Polare*	-44, -10, -12	3.45	33
Reward prediction error at feedback	Ventral striatum (L, R) vmPFC Dorsal striatum (R)	-10, 6, -12	6.25	10394
		10, 10, -8		
		-4, 44, -12		
		16, 6, 16		
	PCC	4, -36, 38	3.66	315
	Superior lateral occipital cortex	-18, -60, 46	5.07	267
	Anterior STS	58, -4, -10	4.67	262
Expertise at decision	PCC/precuneus, ACCg	6, -44, 20	3.77	1458
		2, 14, 30		
	rmPFC	10, 56, 4	4.39	975
	dlPFC	-30, 42, 20	4.05	465
Simulation-based expertise prediction error at observed agent choice	PMv, PMd	44, 12, 16	3.91	1117
		44, 8, 46		
	rTPJ	52, -58, 34	3.99	845
	dmPFC	10, 38, 34	4.05	608
	rSTS/MTG	60, -34, -10	4.18	475
Evidence-based expertise prediction error at feedback	Lateral precuneus	-20, -56, 28	3.95	686
	rdlPFC	28, 48, 26	3.73	646
Differential evidence-based aPEs at feedback: [(AC – DC) – (AI – DI)]*PEOPLE – [(AC – DC) – (AI –DI)]*ALGORITHMS	lOFC	-30, 44, -12	3.55	542
	mPFC	14, 44, 4	3.33	474

Table S2. Contrast, region exhibiting significant effects, MNI coordinates, maximum Z-statistic, and cluster extent for contrasts of interest. Asterisked effects did not survive cluster-based thresholding but survived voxel-wise thresholding at $Z>3.1$, $p<0.001$ (extent greater than 10 voxels). For the remaining contrasts, other than the reward prediction error, we applied a cluster-forming threshold of $Z=2.3$, and a family-wise false positive rate of $p=0.05$. Due to the strength of effects (in height and extent) from the reward prediction error contrast, we applied a cluster-forming threshold of $Z=3.1$, and a family-wise false positive rate of $p=0.01$.

Contrast	Region	Peak Coordinates (MNI)	Z-max	Extent
Sequential model fit on expertise contrast	rmPFC, dmPFC	0, 46, 44	5.09	4680
		8, 58, -2		
	PCC	-6, -22, 28	3.58	577
Sequential model fit on simulation-based aPE contrast	rTPJ*	48, -66, 30	3.82	85
Sequential model fit on evidence-based aPE contrast	Temporo-occipital cortex	-58, -58, -22	4.40	4355
	PMd	-20, 2, 58	4.60	1453
	rdlPFC	30, 46, 24	4.56	815
	rCaudate	18, 20, 0	3.93	423
	ldlPFC	-34, 38, 20	3.97	408
Sequential relative to evidence model fit on simulation-based aPE contrast	Occipital pole, rdlPFC, Temporo-occipital cortex	-10, -98, -19	3.76	689
		30, 44, 24	4.30	550
		-58, -56, -24	4.74	432

Table S3. Contrast, region, MNI coordinates, maximum Z-statistic, and cluster extent for between-subject contrasts of interest. Asterisked effect in rTPJ did not survive cluster-based thresholding across the whole brain but survived small volume correction (see Results). For the table voxel-wise thresholding is applied to rTPJ at $Z>3.1$, $p<0.001$ (extent greater than 10 voxels). For the remaining contrasts, we applied a cluster-forming threshold of $Z=2.3$ and a family-wise false positive rate of $p=0.05$.

SUPPLEMENTAL EXPERIMENTAL PROCEDURES

Bayesian asset tracking model. The basic idea is as follows. Every trial t the subject observes an outcome for the asset y_t. Her problem is to use the history of observations $y_{0:t}$ to estimate the probability that the value of the asset in trial t+1, denoted by q_{t+1}, will go up. The model assumes that the agent estimates this probability using full Bayesian learning (Bayes and Price, 1763) based on the following model of the environment. The asset is assumed to have a Markovian structure described by the following two equations:

$$p(q_{t+1} \mid q_t , v_t) = \mathrm{Beta}(q_t , exp(v_t))$$

and

$$p(v_{t+1} \mid v_t , k) = \mathrm{Normal}(v_t , exp(k)).$$

The first equation describes the stochastic evolution of the asset returns. Here, v_t denotes a volatility parameter measuring the local stability or inertia of the probability parameter: when v_t is high they change quickly, when it is low they don't change much. Importantly, as shown in the second equation, the volatility parameter itself evolves as a Markovian process. Here, the k parameter controls the rate of change in volatility. Note that we use a re-parametrised version of the beta distribution that helps with the numerical computations required to estimate the model, and that also provides the additional intuition about the parameters of the learning model described above. The joint distribution $p(r_{i+1}, v_{i+1}, k)$, which is stored between trials, is set to be uniform at the beginning of the experiment, reflecting the fact that subjects have no information at the onset of the experiment (see (Behrens et al., 2007)).

By applying Bayes rule to this model we can compute the posterior probabilities over the asset parameters, for any history of observed outcomes $y_{1:t}$. In condition 3, the model assumes that the subject makes asset predictions a_t according to the following soft-max function:

$$P(a = up) = \frac{1}{1 + \exp(-\beta(mean(p) - 0.5))}$$

where β is a subject-specific free parameter that reflects the sensitivity of subject choices to asset probability and expertise (see below).

Ability Tracking: Full Bayesian model. Bayes rule can be used to represent the joint probability distribution of the unknowns: the asset *predictability* (r_t = abs(p_t -0.5)) and the agent's ability (α). This belief distribution is conditional on the data: past observations of asset outcomes ($y_{t\leq n}$) and the correctnesss of the agent's previous guesses ($c_{t\leq n}$). Using Bayes rule, we have the following representation of the posterior distribution of the unknowns given the data (note that, to simplify the notation, we have left out reference to v and k):

$$p(r_{t\leq n},\alpha \mid y_{t\leq n},c_{t\leq n}) \propto p(r_1,\alpha)\prod_{t=2}^{n} p(c_t \mid \alpha,r_t)p(y_t \mid r_t)p(r_t \mid r_{t-1}) \quad (1)$$

Integrating over the realizations of r prior to trial n gives the posterior distribution:

$$p(r_n,\alpha \mid y_{t\leq n},c_{t\leq n}) \propto \int p(r_1,\alpha)\prod_{t=2}^{n} p(c_t \mid \alpha,r_t)p(y_t \mid r_t)p(r_t \mid r_{t-1})dr_{t\leq n-1} \quad (2)$$

A similar argument for trial n+1 gives the expression

$$p(r_{n+1},\alpha \mid y_{t\leq n+1},c_{t\leq n+1}) \propto \int p(r_1,\alpha)\prod_{t=2}^{n+1} p(c_t \mid \alpha,r_t)p(y_t \mid r_t)p(r_t \mid r_{t-1})dr_{t\leq n} \quad (3)$$

which, substituting (2) in (3), leads to the *online* update equation for the model given by

$$p(r_{n+1},\alpha \mid y_{t\leq n+1},c_{t\leq n+1}) \propto p(c_{n+1} \mid \alpha,r_{n+1})p(y_{n+1} \mid r_{n+1})\int p(r_{n+1} \mid r_n)p(r_n,\alpha \mid y_{t\leq n},c_{t\leq n})dr_n \quad (4)$$

Marginalizing over r_n we then get

$$p(r_{n+1},\alpha \mid y_{t\le n+1},c_{t\le n+1}) \propto p(c_{n+1} \mid \alpha,r_{n+1})p(y_{n+1} \mid r_{n+1})p(r_{n+1},\alpha \mid y_{t\le n},c_{t\le n}) \tag{5}$$

To make this model tractable, we assume that

$$p(r_{n+1},\alpha \mid y_{t\le n},c_{t\le n}) = p(r_{n+1} \mid y_{t\le n})p(\alpha \mid y_{t\le n},c_{t\le n}). \tag{6}$$

In other words, we assume that c is independent of the future predictability of assets. Substituting in (5) we have

$$p(r_{n+1},\alpha \mid y_{t\le n+1},c_{t\le n+1}) \propto p(c_{n+1} \mid \alpha,r_{n+1})p(y_{n+1} \mid r_{n+1})p(r_{n+1} \mid y_{t\le n})p(\alpha \mid y_{t\le n},c_{t\le n}). \tag{7}$$

Since according to Bayes rule

$$p(r_{n+1} \mid y_{t\le n+1}) \propto p(y_{n+1} \mid r_{n+1})p(r_{n+1} \mid y_{t\le n}),$$

we arrive at our approximation of the full model

$$p(r_{n+1},\alpha \mid y_{t\le n+1},c_{t\le n+1}) \propto p(c_{n+1} \mid \alpha,r_{n+1})p(r_{n+1} \mid y_{t\le n+1})p(\alpha \mid y_{t\le n},c_{t\le n}). \tag{8}$$

The likelihood function $p(c_{n+1}|\alpha,r_{n+1})$ is the generative model that describes the probability distribution of (or belief in) a correct agent guess on trial n+1, given α and r_{n+1}. It is combined with the prior beliefs of α and r to update them and arrive at the posterior beliefs of α and r. For our task,

$$p(c_{n+1}=1|\alpha,r_{n+1}) = \alpha r_{n+1}+(1-\alpha)(1-r_{n+1})$$

and

$$p(c_{n+1} = 0|\alpha,r_{n+1}) = \alpha(1-r_{n+1})+ (1-\alpha)\, r_{n+1}.$$

Reinforcement learning models. We also considered several alternative models related to the pure evidence, pure simulation, and sequential models. In particular, we estimated and compared simple reinforcement-learning implementations of each of the three Bayesian models using a Rescorla-Wagner learning rule (Rescorla and Wagner, 1972). All of these RL models included either a subject-specific parameter for the learning rate about both types of agents, or separate learning rates for people and algorithms. We refer to these models as RL evidence, RL simulation, and RL sequential. In each case, the outcome parameters driving the reinforcement algorithm are based on the g_t variable described above. One version of these has two free parameters (referred to as 2P in Table 1) per subject – a learning rate for all agents and the choice sensitivity term in the softmax function. Another version had three free parameters (3P) per subject – a learning rate for people, a learning rate for algorithms, and the choice sensitivity term in the softmax. A third version had four free parameters (4P) – a learning rate for people, a learning rate for algorithms, a learning rate for the asset, and the choice sensitivity term in the softmax. The performance of these models is shown in main Table 1. None of them performed as well as their Bayesian counterparts.

Behavioral regression analyses. We first predicted subject bets for or against human (v_t^p) and algorithm (v_t^a) agents separately on the basis of correct and incorrect agent predictions over the previous five trials with that agent. This lead to the following regression equations for people

$$v_t^p = \beta_1 c_{t-1}^p + \beta_2 c_{t-2}^p + \beta_3 c_{t-3}^p + \beta_4 c_{t-4}^p + \beta_5 c_{t-5}^p$$

and for algorithms

$$v_t^a = \beta_1 c_{t-1}^a + \beta_2 c_{t-2}^a + \beta_3 c_{t-3}^a + \beta_4 c_{t-4}^a + \beta_5 c_{t-5}^a ,$$

where c_t corresponds to correct ($c_t = 1$) or incorrect ($c_t = 0$) agent predictions on trial t.

We next divided correct and incorrect agent predictions into those with which subjects agreed or disagreed, on the basis of their inferred beliefs from the asset-tracking model. This resulted in four binary independent regressors for each of the five previous interactions (20 regressors in total) which we used to predict subject votes on the current trial: interactions on which subjects likely agreed when the agent was correct (AC), agreed when the agent was incorrect (AI), disagreed when the agent was correct (DC), and disagreed when the agent was incorrect (DI). More specifically, we predicted subject bets for or against people or algorithms on the current trial in two separate models (one for people and one for algorithms) using the following binary predictor matrix: AC_{t-1}, AI_{t-1}, DC_{t-1}, DI_{t-1}, AC_{t-2}, AI_{t-2}, DC_{t-2}, DI_{t-2}, AC_{t-3}, AI_{t-3}, DC_{t-3}, DI_{t-3}, AC_{t-4}, AI_{t-4}, DC_{t-4}, DI_{t-4}, AC_{t-5}, AI_{t-5}, DC_{t-5}, DI_{t-5}. To perform statistical comparisons, we computed the arithmetic means of the regression coefficients from these models separately for people and algorithms, pooling coefficients from regressors of the same type ($AC_{t-1:t-5}$, $AI_{t-1:t-5}$, $DC_{t-1:t-5}$, $DI_{t-1:t-5}$). In each of these regressions (people and algorithms) there were 88 choice trials and only 20 independent variables. Furthermore the statistical tests were carried out on the averages across groups of 5 of these independent variables (t-1 to t-5). The variance of the estimates of the key variables of interest were therefore equivalent to a test with 88 timepoints and only 4 independent variables (see (Smith et al., 2007) for variance computations on linear combinations of parameter estimates in GLMs). These regression coefficients and their means are plotted in Figure 3.

We note that the interaction effect remains significant if we instead use only the last trial (t -1; p=0.04), but the effect is notably stronger when using the mean across the past five trials (p=0.006). Also, the FMRI results reported in Figure 7 and S4 do not depend on whether the last trial only or the mean of the past five trials are used to explain behavior.

Regressors for computational fMRI analysis. In addition to testing which of these models fit the subject's behavior best, we also look for evidence consistent with their neural implementation. This was done using the parametric general linear model (GLM) of BOLD responses described below, which makes use of the following parametric

regressors. All of them were computed based on the fits of the Bayesian sequential model (described in the Methods section of the main text) that describes the subjects' choices best. First, the model looks for correlates of estimated agent ability at the beginning of trials, which is measured by α_t. Second, the model looks for neural correlates of unsigned ***ability prediction errors (aPEs)***, which are given by

$$\begin{cases} abs(1-\alpha_t) & \text{when } g_t\text{=1} \\ abs(-\alpha_t) & \text{when } g_t\text{=0} \end{cases}$$

where *abs* denotes the absolute value operator. We test for aPEs at two different timepoints within the trial: A) ***simulation-based aPEs*** time-locked to the revelation of the agent's prediction, which is the time when subjects can first infer whether or not the agent made a good prediction on that trial based on their own beliefs., and B) ***evidence-based aPEs*** time-locked to the feedback period, which is the time at which subjects first discover whether or not the agent's prediction on that trial was correct. Evidence-based aPEs are computed with respect to the new prior over α_t, which was updated on the basis of the agent's most recent prediction. The model also tests for correlates of ***expected reward value (EV)*** of the choice, which in condition 3 is defined by

$$\begin{cases} mean(p_t) & \text{when } a_t\text{=1} \\ 1-mean(p_t) & \text{when } a_t\text{=0} \end{cases}$$

where a_t=1 denotes a prediction by the agent that the asset will increase in value on trial *t*. In conditions 1 and 2, the expected reward value of the choice is defined by

$$\begin{cases} \alpha_t & \text{when } b_t\text{=1} \\ 1-\alpha_t & \text{when } b_t\text{=0} \end{cases}$$

where b_t=1 denotes a bet for the agent on trial *t*. Finally, the model also tests for ***reward prediction errors (rPEs)*** at feedback, based on the outcomes of subjects' predictions. Let o_t denote the outcome of the subject's prediction on trial t. On every trial, either o_t =1, if the subject guessed correctly, or o_t=0 otherwise. rPEs were therefore given by

$$o_t - EV_t.$$

Note that, in contrast to aPEs, the rPEs are signed.

FMRI Data Acquisition. FMRI data were acquired on a Siemens 3T TIM-TRIO full body scanner using a Siemens 32-channel phased array head coil. We acquired high-resolution anatomical images using a T1-weighted protocol (FOV, 256; 176 slices, 1 × 1 × 1 mm). Functional imaging used a gradient echo EPI sequence (repetition time (TR)=2530 ms; Echo time (TE)=30 ms; field of view (FOV)=192; anterior-to-posterior phase encoding, ascending slice acquisition). We acquired four functional runs, each with 40 oblique axial slices aligned 30° off the AC–PC plane to reduce signal dropout in the orbitofrontal cortex (Deichmann et al., 2003), which has previously been implicated in aspects of reinforcement learning and decision-making, 3mm isometric voxels and a 0.3mm inter-slice gap. Stimulus presentation/subject button presses were registered and time-locked to FMRI data using *Presentation* (Neurobehavioural Systems, USA).

FMRI data preprocessing. Data were preprocessed using the default options in FMRIB's Software Library (FSL)(Jenkinson et al., 2011): motion correction was applied using rigid body registration to the central volume (Jenkinson et al., 2002); Gaussian spatial smoothing was applied with a full width half maximum of 5mm; brain matter was segmented from non-brain using a mesh deformation approach (Smith, 2002); high pass temporal filtering was applied using a Gaussian-weighted running lines filter, with a 3dB cutoff of 100s.

FMRI analyses. For group analyses, EPI images were first registered to the high resolution structural image using 6 degrees of freedom and then to the standard (Montreal Neurological Institute) space MNI152 template using affine registration with 12 degrees of freedom (Jenkinson and Smith, 2001). We then fit a GLM to estimate the group mean effects for the regressors described above. For expertise and expertise prediction error COPEs, we also included a between-subject covariate reflecting the relevant behavioral index in the group-level GLM in addition to the mean. FMRIB's Local Analysis of

Mixed Effects (FLAME) was used to perform a mixed effects group analysis (Beckmann et al., 2003; Woolrich et al., 2004). All reported fMRI Z-statistics and p-values arose from these mixed effects analyses on all 25 subjects. Unless otherwise stated, we report significant effects at a cluster-forming threshold across the whole brain of Z=2.3, and a family-wise false positive rate of p=0.05.

ROI analyses. We used a leave-one-out extraction procedure to provide an independent criterion for voxel selection (Kriegeskorte et al., 2009). In each participant, BOLD signal was extracted from a 3mm radius sphere centered on the peak voxel for each contrast of interest in a group model excluding that participant. We then performed independent statistical tests and characterized the time course of BOLD fluctuations in these regions for trials with people and algorithms separately.

To produce time-course plots, each subject's BOLD timeseries for a given region was then divided into trials, which were upsampled by (TR=3000ms)/10 to 300ms using a spline interpolation. Trials were then truncated based on the mean trial length for each condition across trials and subjects. This resulted in a data matrix of size trials x mean trial length. A general linear model was fit using ordinary least squares across trials at each time point in each subject independently. We then calculated group mean effect sizes at each time point, and their standard errors. Data and regressors were Z-normalized (we removed the mean and made the std=1) so that effect sizes could be reported as (partial) correlations.

SUPPLEMENTAL TEXT

Relative fit of ability tracking models. Although the sequential model provided the best fit on average (in 18/25 subjects), there was nonetheless inter-individual variability in the differential fit between models (see Fig. S1). In particular, the evidence model best described choices in 4/25 subjects, the simulation model in 3/25 subjects, and the sequential model in 18/25 subjects (Fig. S1). This variability is exploited in some of the neural analyses reported.

People vs Algorithms. We compared subject behavior when tracking people and algorithms using several other measures, and obtained similar results. The mean negative log likelihood (NlogL) of the sequential model was 49.24 (SD=9.58) for algorithms and 49.69 (SD=10.04) for people (paired t-test for difference between algorithms and people: t(24)=-0.27, p=0.39). Similar results were observed when instead analyzing the relative fit of the sequential compared to the evidence model. The mean difference in NlogL between sequential and evidence models was 1.06 (SD=6.27) for algorithms and 1.80 (SD=6.27) for people (t(24): =-0.38, p=0.36). Although the sequential model explained choices far better than the simulation model for both people and algorithms (see Fig. S1), there was a trend toward a better relative fit of the sequential compared to the simulation models in people compared to algorithms (t(24)=1.5, p=0.07). In addition to NlogL, we also compared maximum likelihood estimates (MLEs) for the choice sensitivity term β and learning rate λ, which was estimated in the RW variant of the sequential Bayesian model. MLEs for β from the sequential model were similar for algorithms (mean NlogL=4.41, SD=3,11) and people (mean=4.12, SD=2.81; t(24)=0.78, p=0.22). MLEs for λ were also similar for algorithms (mean=0.011, SD=0.13) and people (mean=0.093, SD=0.13; t(24)=0.23, p=0.41). The amount of money earned when predicting the accuracy of people and algorithms also did not differ statistically. On average subjects earned \$8.84 (SD=\$12.59) for interactions with human agents and \$9.12 (SD=\$13.0) for interactions with algorithms (t(24)=-1.0, p=0.15).

Choice frequency analysis. For robustness, we have also computed choice frequencies following each of the different outcome types of interest (AC, DC, AI, and DI) at previous trial *n*-1, which are shown in Fig. S2. Using this approach we found that subjects bet for people significantly more frequently than for algorithms following AC trials (two-tailed one-sample t-test: $t(24)=2.20$, $p<0.05$), and less frequently following AI trials ($t(24)=2.16$, $p<0.05$), with no significant difference on DC and DI trials (all $t(24)<0.5$, $p>0.1$). Furthermore, the probability of betting for people on AC compared to DC trials was marginally greater for people than algorithms ($t(24)=1.58$, $p=0.06$), and the probability of betting against algorithms was significantly greater than for people on AI trials compared to DI trials ($t(24)=1.99$, $p<0.05$). The difference between AC-DC on betting for the agent and AI-DI on betting against the agent was also strongly significant ($t(24)=3.73$, $p<0.0001$). These choice frequency analyses thus support the findings of the regression analyses. They indicate that agreeing with the agent's prediction increases subjects' likelihood of voting for people compared to algorithms following correct predictions and decreases their likelihood of voting against people compared to algorithms following incorrect predictions.

Fixed effects behavioral model. To directly relate the regression and model-based analyses, we constructed an additional reinforcement-learning model with a Rescorla Wagner update rule that allowed for the possibility of differential updating on AC, DC, AI, and DI trials for people and algorithms. The ability levels for people (α^p_t) and algorithms (α^a_t) are given by

$$\alpha^p_t=\alpha^p_{t-1}+\gamma^p(c_{t-1}-\alpha^p_{t-1}) \text{ if } AC_{t-1}=1$$

$$\alpha^p_t=\alpha^p_{t-1}+\eta^p(c_{t-1}-\alpha^p_{t-1}) \text{ if } DC_{t-1}=1$$

$$\alpha^p_t=\alpha^p_{t-1}+\varphi^p(c_{t-1}-\alpha^p_{t-1}) \text{ if } AI_{t-1}=1$$

$$\alpha^p_t=\alpha^p_{t-1}+\lambda^p(c_{t-1}-\alpha^p_{t-1}) \text{ if } DI_{t-1}=1$$

for people, and by

$$\alpha^{a}_{t}=\alpha^{a}_{t-1}+\gamma^{a}(c_{t-1}-\alpha^{a}_{t-1}) \text{ if } AC_{t-1}=1$$

$$\alpha^{a}_{t}=\alpha^{a}_{t-1}+\eta^{a}(c_{t-1}-\alpha^{a}_{t-1}) \text{ if } DC_{t-1}=1$$

$$\alpha^{a}_{t}=\alpha^{a}_{t-1}+\varphi^{a}(c_{t-1}-\alpha^{a}_{t-1}) \text{ if } AI_{t-1}=1$$

$$\alpha^{a}_{t}=\alpha^{a}_{t-1}+\lambda^{a}(c_{t-1}-\alpha^{a}_{t-1}) \text{ if } DI_{t-1}=1$$

for algorithms. As with the other models, bets were modeled with the softmax function:

$$P(b_t = for) = \frac{1}{1 + \exp(-\beta(mean(\alpha_t) - 0.5))},$$

Relationship between aPEs and Bayesian updating. Supplemental Figure S7 plots the relationship between our neural marker of ability updating (i.e., the unsigned aPEs) and the Bayesian updates generated by the sequential model (i.e., $\alpha_t - \alpha_{t-1}$). These variables were highly correlated (mean $r = 0.74$) and were therefore not dissociable with our task. Note that the Bayesian update term is generally slightly larger early during the learning about a new agent, and slightly smaller later on. We used the unsigned aPEs as our neural marker of ability updating, rather than full the Bayesian updates, because there is evidence that prediction errors are encoded separately from attentional signals that may determine the weighting on those prediction errors that together can be seen to produce the full update term (Behrens et al., 2008; Roesch et al., 2012). However, the current study cannot determine if the identified neural signals reflect the attentional weighting proposed by such models, or the prediction errors themselves.

SUPPLEMENTAL REFERENCES

Bayes, T., and Price, R. (1763). An Essay towards solving a Problem in the Doctrine of Chance. By the late Rev. Mr. Bayes, communicated by Mr. Price, in a letter to John Canton, A. M. F. R. S. Philosophical Transactions of the Royal Society of London *53*, 370-418.

Beckmann, C.F., Jenkinson, M., and Smith, S.M. (2003). General multilevel linear modeling for group analysis in FMRI. Neuroimage *20*, 1052-1063.

Behrens, T.E., Hunt, L.T., Woolrich, M.W., and Rushworth, M.F. (2008). Associative learning of social value. Nature *456*, 245-249.

Behrens, T.E., Woolrich, M.W., Walton, M.E., and Rushworth, M.F. (2007). Learning the value of information in an uncertain world. Nat Neurosci *10*, 1214-1221.

Deichmann, R., Gottfried, J.A., Hutton, C., and Turner, R. (2003). Optimized EPI for fMRI studies of the orbitofrontal cortex. Neuroimage *19*, 430-441.

Jenkinson, M., Bannister, P., Brady, M., and Smith, S. (2002). Improved optimization for the robust and accurate linear registration and motion correction of brain images. Neuroimage *17*, 825-841.

Jenkinson, M., Beckmann, C.F., Behrens, T.E., Woolrich, M.W., and Smith, S.M. (2011). Fsl. Neuroimage.

Jenkinson, M., and Smith, S. (2001). A global optimisation method for robust affine registration of brain images. Med Image Anal *5*, 143-156.

Kriegeskorte, N., Simmons, W.K., Bellgowan, P.S., and Baker, C.I. (2009). Circular analysis in systems neuroscience: the dangers of double dipping. Nat Neurosci *12*, 535-540.

Roesch, M.R., Esber, G.R., Li, J., Daw, N.D., and Schoenbaum, G. (2012). Surprise! Neural correlates of Pearce-Hall and Rescorla-Wagner coexist within the brain. The European journal of neuroscience *35*, 1190-1200.

Smith, S., Jenkinson, M., Beckmann, C., Miller, K., and Woolrich, M. (2007). Meaningful design and contrast estimability in FMRI. Neuroimage *34*, 127-136.

Smith, S.M. (2002). Fast robust automated brain extraction. Hum Brain Mapp *17*, 143-155.

Woolrich, M.W., Behrens, T.E., Beckmann, C.F., Jenkinson, M., and Smith, S.M. (2004). Multilevel linear modelling for FMRI group analysis using Bayesian inference. Neuroimage *21*, 1732-1747.
